# A bacterial actin with high ATPase activity regulates the polymerization of a partner MreB isoform essential for *Spiroplasma* swimming motility

**DOI:** 10.1016/j.jbc.2025.110462

**Published:** 2025-07-07

**Authors:** Daichi Takahashi, Hana Kiyama, Hideaki T. Matsubayashi, Ikuko Fujiwara, Makoto Miyata

**Affiliations:** 1 Graduate School of Science, Osaka Metropolitan University, Osaka, Japan; 2 Frontier Research Institute for Interdisciplinary Sciences, Tohoku University, Miyagi, Japan; 3 Department of Materials Science and Bioengineering, Nagaoka University of Technology, Nagaoka, Niigata, Japan; 4 The OMU Advanced Research Center for Natural Science and Technology, Osaka Metropolitan University, Osaka, Japan

**Keywords:** ATPase, Bacterial Actin, Cytoskeletal Crosstalk, Polymerization Dynamics, Membrane Binding

## Abstract

*Spiroplasma* is a wall-less helical bacterium that is characterized by a unique swimming motility involving five isoforms of the bacterial actin MreBs (SMreB1–5). The functions of SMreBs are unique in the MreB family proteins, as their counterparts in walled bacteria localize the cell wall synthesis complex by forming filaments that slowly turn over to maintain the cell shape. *In vitro* analyses of individual SMreBs provide clues to understand the detailed molecular mechanism of *Spiroplasma* swimming. However, the purification difficulties have hampered *in vitro* analyses of one of the SMreBs, SMreB1, which is essential for the swimming. Here, we isolated soluble SMreB1 of *Spiroplasma eriocheiris* (SpeMreB1) and evaluated its activity. SpeMreB1 was expressed as a fusion with a solubilization tag, ProteinS, which allowed us to purify it in the soluble fraction. SpeMreB1 exhibited the highest phosphate (P_i_) release rate and the fold changes of critical concentrations for polymerization across the nucleotide states among the MreB family proteins. SpeMreB1 interacted with polymerized SpeMreB5, another SMreB essential for *Spiroplasma* swimming. In the AMPPNP- or ADP-bound state, SpeMreB1 decreased the amount of SpeMreB5 filaments, possibly reflecting their disassembly. Regardless of the nucleotide state, SpeMreB1 bound to negatively charged lipids. These results suggest that SpeMreB1 utilizes its highest activity to manage SpeMreB5 filaments underneath the cell membrane to drive *Spiroplasma* swimming.

Motility is the ability of an organism to move from one starting point to another using mechanical energy (converted from chemical energy). Because this ability plays an important role in the survival of organisms, such as obtaining nutrients and evading predators, the evolution of motility has been linked to that of organisms themselves (1). In most bacteria, the peptidoglycan layer, the bacterial cell wall, plays an important role for their motility. The most conserved bacterial motility machineries, flagella and pili (2), require the anchor of the peptidoglycan layer for their propulsion (1). However, because of its conservation across the bacterial kingdom, the peptidoglycan layer is often targeted by natural immunity (3, 4). Therefore, the absence of peptidoglycans is likely advantageous to pathogenic bacteria (1, 5). Certain peptidoglycan-deficient species have evolved unique motility systems that do not rely on conventional bacterial motility machineries requiring the peptidoglycan layer (1).

*Spiroplasma* is a wall-less helical bacterium pathogenic to plants and arthropods. It is often considered industrially problematic, as certain species exhibit strong pathogenicity to host organisms (6, 7, 8, 9). *Spiroplasma* swims by propagating its cell helicity switching along the cell axis to generate a propulsive force (10, 11, 12). Each *Spiroplasma* species possesses five isoforms of bacterial actin proteins MreB (SMreB1–5), which contribute to swimming motility (13, 14, 15, 16, 17). A recent study suggested that the swimming motility of *Spiroplasma* is related to its pathogenicity (6, 18), as does the motility of other pathogenic organisms (1). Studies of SMreBs are therefore important not only for elucidating the molecular mechanism of *Spiroplasma* swimming but also for understanding its pathogenic process.

Although MreB is conserved in many bacteria, particularly those with elongated cell shapes and cell walls, its well-known role is related to maintaining cell shape rather than contributing to dynamic motility (19). In walled-bacteria, MreB filaments bind to the cell membrane and support the formation and localization of a peptidoglycan synthesis complex for the cell elongation (elongasome complex) (20, 21). As peptidoglycan synthesis progresses, MreB filaments move along the cell membrane in a direction perpendicular to the major cell axis. This movement is driven by the peptidoglycan synthesis itself, rather than by the polymerization dynamics of MreB. Thus, MreB filaments are believed to be static during the cell shape maintenance process (22, 23). The static nature of MreB is also suggested by its small conformational change upon polymerization compared with other actin superfamily proteins (24). It is an open question how a dynamic phenomenon like *Spiroplasma* swimming could have emerged in MreB family proteins, which are supposed to be static in other bacterial cells.

Because of its importance in pathogenicity and the evolutionary interest, *Spiroplasma* swimming has been studied from several perspectives, including cellular and molecular biology (25). A previous study using JCVI-syn3B, a minimal synthetic bacterium, revealed that a combination of SMreB5 and either the phylogenetically related SMreB1 or SMreB4 is essential for *Spiroplasma* swimming (14). SMreB5 has been studied biochemically and structurally, and the following properties have been reported (16, 26, 27, 28): SMreB5 possesses ATPase and polymerization activities; and SMreB5 filaments are suggested to be metastable in the ADP-P_i_-bound state and primed to depolymerize after the P_i_ release—features reminiscent of eukaryotic actin. SMreB5 also forms asymmetric sheets composed of an antiparallel double-stranded filament common to the studied MreBs and parallel-oriented protofilaments; SMreB5 binds to a negatively charged membrane mimicking the *Spiroplasma* cell *via* its positively charged and unstructured C-terminal tail; and SMreB5 forms bundles depending on its ATPase activity and surface charge. However, SMreB1 and SMreB4 have not been studied because they were not purified in the soluble fraction (27).

In the present study, we solubilized SMreB1 and analyzed it using biochemical techniques. SMreB1 of the crustacean pathogen *Spiroplasma eriocheiris* (7) (SpeMreB1) was fused with ProteinS (PrS), which is a spore surface protein of a soil bacterium *Myxococcus xanthus* and has also been reported as a solubilization tag (29). Soluble SpeMreB1 was investigated for its activity, crosstalk with SpeMreB5, and its membrane-binding ability. Based on these analyses, we discuss the functions of SpeMreB1.

## Results

### Preparation of PrS-fused soluble SpeMreB1

**Figure 1:**
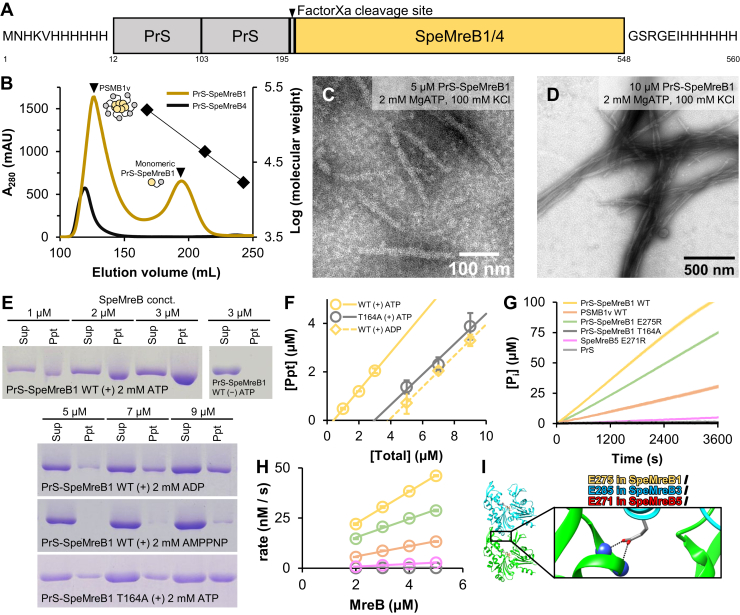
**Preparation and evaluation of PrS-SpeMreB1 and PrS-SpeMreB4.***A,* domain architecture of PrS-fused SpeMreB1 and SpeMreB4. The N termini of SpeMreB1 and SpeMreB4 were fused with a 6× His-tag, two consecutive PrS molecules, and a factor Xa cleavage site. The C termini of SpeMreB1 and SpeMreB4 were fused with a 6× His-tag. The numbers underneath the figure indicate the residue numbers of PrS-SpeMreB1. *B,* gel filtration profiles of PrS-SpeMreB1 WT (*dark yellow*) and PrS-SpeMreB4 WT (*black*). Peak positions of PSMB1v WT and the monomeric PrS-SpeMreB1 WT are indicated by *closed triangles*. Bovine γ-globulin (158 kDa), chicken ovalbumin (44 kDa), and horse myoglobin (17 kDa) were used as the protein size standards, and their elution volumes are indicated by closed diamonds with the linear fit over the log of their molecular weights. *C* and *D,* negative-staining EM images of (*C*) 5 μM and (*D*) 10 μM PrS-SpeMreB1 WT in the standard buffer (20 mM Tris–HCl [pH 7.5], 100 mM KCl, 5 mM DTT, 2 mM MgCl_2_, and 2 mM ATP). *E,* sedimentation assays of PrS-SpeMreB1 WT over the nucleotide states and PrS-SpeMreB1 T164A in the presence of ATP. *F,* quantification of pellet amounts of PrS-SpeMreB1 WT in the presence of ATP or ADP and PrS-SpeMreB1 T164A in the presence of ATP. The resulting concentrations of the precipitated fractions were plotted against the total SpeMreB concentrations with linear fitting. Error bars indicate the SD of three independent measurements. Critical concentrations were estimated as the *x*-intercept of each linear fit and are summarized in Table 1. *G* and *H,* (*G*) time course and (*H*) concentration dependence of P_i_ release from PrS-SpeMreB1 WT (*yellow*), PSMB1v WT (*orange*), PrS-SpeMreB1 E275R (*light green*), PrS-SpeMreB1 T164A (*dark gray*), SpeMreB5 E271R (*pink*), and PrS (*light gray*). Data are presented as mean ± SD of three independent measurements. *G,* protein concentrations were set at 3 μM for SpeMreBs and 5 μM for PrS. P_i_ release rates (*k*_Pi_) were estimated from the slopes of the linear fits in *H* and are summarized in Table 2. *I,* ribbon representation of two consecutive molecules along the *a*-axis in the crystal of the SpeMreB3–AMPPNP complex (Protein Data Bank: 7E1G) and the close-up view of its intraprotofilament interaction region. Residue E285 is represented using the stick model with the corresponding residue numbers in SpeMreB1, SpeMreB3, and SpeMreB5.

As previously reported (27), neither the 6× His-tag fused SpeMreB1 nor SpeMreB4 was soluble (Fig. S1*A*). This was also the case for SMreB1 and SMreB4, which were derived from different *Spiroplasma* species (Fig. S1, *B* and *C*, Data S2). We coexpressed SpeMreB4 and SpeMreB5 because the genes for expressing them are coded tandemly in the genomes of many *Spiroplasma* species (17, 30). However, this approach did not solve the insolubility problem of SpeMreB4 (Fig. S1*D*). Therefore, we used a solubilization tag for SpeMreB1 and SpeMreB4. We fused two consecutive PrS molecules at the N termini of SpeMreB1 and SpeMreB4 (PrS-SpeMreB1 and PrS-SpeMreB4, respectively) and a 6× His-tag at their C termini (Fig 1*A*, Data S1) and successfully purified them using Ni^2+^–NTA affinity chromatography and gel filtration (Fig. S1*E*). During gel filtration, all PrS-SpeMreB4 molecules were eluted in the void fraction where the molecular size exceeded the upper separation limit of the column, indicating that PrS-SpeMreB4 formed oligomers. In contrast, PrS-SpeMreB1 was eluted in both the void and monomeric fractions (Fig. 1*B*). The oligomer formation of PrS-SpeMreB1 and PrS-SpeMreB4 was probably not caused by the soluble aggregation (aggregate but not precipitated by standard centrifugation) of PrS, as is reported with other solubilization tags (31) because a gel filtration detected the PrS carrying the 6× His-tags at both the N and C termini, which was used as the negative control in the following experiments, at the monomeric fraction even at a high concentration for the purification and the following assays (50 μM) (Fig. S2*A*). In this study, we retained the PrS tag on these SpeMreBs, because the tag removal by factorXa precipitated SpeMreB1 (Fig. S1*F*).

### Polymerization of SpeMreB1 is highly dynamic among MreB family proteins

We evaluated the assembled structures of PrS-SpeMreB1 after the polymerization of its monomeric fraction by adding ATP. PrS-SpeMreB1 formed sheet structures in the presence of ATP (Fig. 1*C*). Their 2D averaged image showed the 5.0 ± 0.2 nm subunit repeat consistent with other MreB filaments (16, 20, 27, 28, 32, 33, 34), although the protofilament arrangement was obscure probably because of the small particle number available for the image averaging (∼1000 particles per 50 micrographs) (Fig. S3*A*). Increasing the PrS-SpeMreB1 concentration from 5 to 10 μM resulted in the filament bundling (Fig. 1*D*), whereas the bundles were partially disassembled by increasing the salt concentration from 100 to 500 mM (Fig. S3*B*), indicating that the filament bundling of PrS-SpeMreB1 is governed by electrostatic interactions similar to that of SpeMreB5 (26). In contrast, PrS-SpeMreB1 did not polymerize under nucleotide-free (Nf) conditions (Fig. S3, *C* and *D*).

Next, to evaluate the polymerization activity of PrS-SpeMreB1, we performed sedimentation assays across nucleotide states and estimated each critical concentration (C_C_), which is the minimum concentration required for polymerization (Fig. 1, *E* and *F*, Table 1). Although PrS-SpeMreB1 did not precipitate under Nf conditions, in the presence of ATP, it precipitated within the C_C_ range compatible with SpeMreB5. In contrast, PrS-SpeMreB1 was less polymerized in the presence of ADP, and its C_C_ was 9.3-fold higher than that in the presence of ATP. This fold change was larger than the C_C_ differences of SpeMreB5 in the presence of ATP and ADP (2.8-fold). PrS-SpeMreB1 hardly polymerized in the presence of AMPPNP (a nonhydrolyzed ATP analog). The C_C_ of an SpeMreB1 variant carrying the active center for ATP hydrolysis (27), PrS-SpeMreB1 T164A, was 7.3-fold higher than that of the WT in the presence of ATP. Considering the polymerization cycle established by the analyses of SpeMreB3 and SpeMreB5, where the filaments are stabilized by the hydrolysis and depolymerized after the P_i_ release (27), these results suggest that SpeMreB1 filaments exchange their subunits more actively than SpeMreB5 filaments, depending on the change in the nucleotide state.

**Table 1:** Bulk critical concentrations of SpeMreBs estimated from sedimentation assays^a^

Construct-nucleotide	PrS-SpeMreB1 WT-ATP	PrS-SpeMreB1 WT-ADP	PrS-SpeMreB1 WT-AMPPNP	PrS-SpeMreB1 T164A-ATP	SpeMreB5 WT-ATP	SpeMreB5 WT-ADP	SpeMreB5 WT-AMPPNP	SpeMreB5 T160A-ATP
C_C_ (μM)	0.41 ± 0.05	3.8 ± 0.7	≥7	3.0 ± 0.2	0.14 ± 0.07	0.39 ± 0.09	0.29 ± 0.11	0.15 ± 0.15
Reference	This study	This study	This study	This study	Takahashi D *et al.*, 2022 *Open Biol*. 12 (10):220083^27^	Takahashi D *et al.*, 2022 *Open Biol*. 12 (10):220083^27^	Takahashi D *et al.*, 2022 *Open Biol*. 12 (10):220083^27^	Takahashi D *et al.*, 2022 *Open Biol*. 12 (10):220083^27^

a Values are indicated as the mean ± SD of three independent measurements, except for PrS-SpeMreB1 WT in the presence of AMPPNP whose critical concentrations could not be determined by the linear fitting because of the small pellet amounts.

Next, to evaluate the ATPase activity of SpeMreB1, we measured P_i_ release. Although P_i_ release was not detected with PrS alone, SpeMreBs released P_i_ linearly over time (Fig. 1*G*). The P_i_ release rate of PrS-SpeMreB1 was 5.3-fold higher than that of SpeMreB5 (27) (Fig. 1*H*, Table 2) and also 2.9- and 3.1-fold higher than that of *Escherichia coli* MreB, which had the highest record of the P_i_ release rate among the studied MreB family proteins (35) and animal actin (36), respectively. The P_i_ release rate of PrS-SpeMreB1 T164A was 200-fold lower than that of the WT, confirming that SpeMreB1 has an ATP hydrolysis mechanism common to other MreB family proteins, as previously suggested (27). Taken together, these results suggest that the polymerization of SpeMreB1 is highly dynamic among the MreB family proteins.

**Table 2:** P_i_ release rates of SpeMreBs^a^

Construct	PrS-SpeMreB1 WT	PSMB1v WT	PrS-SpeMreB1 E275R	PrS-SpeMreB1 T164A	SpeMreB5 WT	SpeMreB5 E271R	SciMreB5 WT	EcMreB	Actin
k_Pi_ (nM [P_i_]/s/μM [protein])	8.0 ± 0.1	2.5 ± 0.1	4.6 ± 0.1	0.04 ± 0.02	1.5 ± 0.2	0.66 ± 0.04	2.5 ± 0.1	2.8 ± 0.2	2.6 ± 0.4
Reference	This study	This study	This study	This study	Takahashi *et al.*, 2022 *Open Biol*. 12 (10):220083^27^	This study	Pande V. *et al.* 2022 *J Cell Biol.* 221 (5):e20210692^28^	Nurse P. and Marians K.J. (2013). *J. Biol. Chem*. 288 (5):3469-75^35^	Melki R. *et al.* 1996. *Biochemistry*. 35 (37):12038–45^36^

a Values are indicated as the mean ± SD of three independent measurements.

### SpeMreB1 exhibits ATPase activity in both monomeric and polymerized states

The linearity of the time-course P_i_ release measurements suggests a futile cycle in which ATP is consumed by SpeMreB monomers, similar to *Geobacillus stearothermophilus* MreB (GsMreB) (32) (Fig. 1*G*). To confirm this possibility, we created polymerization-deficient variants of SpeMreB1 and SpeMreB5 and measured their P_i_ release rates. Referring to the crystal structure of the SpeMreB3–AMPPNP complex, the E285 residue electrostatically interacts with the positively charged side of the α-helix starting from V216 (27). The corresponding residue of SpeMreB3 E285 is conserved in SMreBs, including SpeMreB1 and SpeMreB5 (E275 and E271 for SpeMreB1 and SpeMreB5, respectively) (15) (Fig. 1*I*). Therefore, we generated PrS-SpeMreB1 E275R and SpeMreB5 E271R as polymerization-deficient variants. These variants were not polymerized as confirmed by sedimentation assays (Fig. S1*G*). PrS-SpeMreB1 E275R and SpeMreB5 E271R still exhibited ATPase activities that were 1.7- and 2.3-fold lower, respectively, than those of the corresponding WTs. The decrease in the P_i_ release rate in the polymerization-deficient variant of SpeMreB5 is consistent with the results of a previous study (28). These results indicate that SpeMreB1 and SpeMreB5 also possess futile cycles similar to those of GsMreB (32) and that these ATPase activities are promoted by polymerization.

### Polymerization of SpeMreB5 is required for the binding between SpeMreB1 and SpeMreB5

Next, we evaluated the interactions between SpeMreB1 and SpeMreB5. However, when different kinds of polymerizing proteins are mixed, it is difficult to distinguish whether the obtained signals (*e.g.*, increasing precipitate amount in sedimentation assays) are due to their interactions or promoted polymerization. Therefore, we used polymerization-deficient SpeMreB1 variants to detect their interactions with SpeMreB5. In addition to the PrS-SpeMreB1 E275R variant, we analyzed the interaction of PrS-SpeMreB1 WT eluted in the void fraction at gel filtration (PSMB1v) (Fig. 1*B*). The size distribution of PSMB1v did not depend on the incubation and the presence of ATP (Fig. S2, *B*–*E*), and it was less precipitated by an ultracentrifugation even in the presence of ATP (Fig. S2*F*). These results suggest that the subunits within the PSMB1v oligomers are not exchanged with other PrS-SpeMreB1 molecules. Nevertheless, PSMB1v retained several characteristics of the PrS-SpeMreB1 monomer: namely; the presence of the ATPase activity, although its P_i_ release rate was 3.2-fold lower than that of the PrS-SpeMreB1 monomer (Table2, Fig. 1, *G* and *H*); and the crosstalk with SpeMreB5 shown in the next paragraph. PSMB1v also retained the structural characteristics of the PrS-SpeMreB1 monomer as estimated by CD measurements (Table 3, Fig. S2*G*). Although the β-strand content of PrS-SpeMreB1 was 8.4% higher than that predicted from its amino acid sequence, 5.5% of the 8.4% is due to the large gap between the theoretical and experimental results for the β-strand content of PrS, which occupies 39.1% of the residues in PrS-SpeMreB1 (14.1% × 0.391 = 5.5%). Excluding this, PrS-SpeMreB1 probably adopted the structure as predicted, because the differences in α-helix and β-strand content of PrS-SpeMreB1 in experiment and prediction remained at 6.3% and 2.9%, respectively. Although the α-helix content of PSMB1v was 4.3% lower than that of PrS-SpeMreB1, possibly reflecting partial denaturation of PSMB1v, the other differences were not detected. Therefore, we used PSMB1v to analyze its interaction with SpeMreB5. In addition to SpeMreB1, we performed sedimentation assays of PrS-SpeMreB4 eluted in the void fraction (Fig. 1*B*). Although PrS-SpeMreB4 precipitated regardless of ATP (Fig. S1*H*), filamentous structures were not formed, even in the presence of ATP (Fig. S3*E*), suggesting that PrS-SpeMreB4 sedimentation was caused by PrS-SpeMreB4 aggregation rather than polymerization. Therefore, we excluded PrS-SpeMreB4 from further analyses.

**Table 3:** Secondary structure contents and number of amino acid residues in PrS-SpeMreB1 and PrS^a^

Constructs	**α**-helix (%)	**β**-strand (%)	Amino acid residues (a.a.)
PrS-SpeMreB1 (CD)	23.2	32.7	560
PSMB1v (CD)	18.9	32.7	
PrS-SpeMreB1 (theoretical)	29.5	24.3	
PrS (CD)	8.1	45.6	219
PrS (theoretical)	8.7	31.5	

a The secondary structure contents in the rows labeled CD and theoretical were estimated from the CD spectra (Fig. S2*G*) and amino acid sequences, respectively.

**Figure 2:**
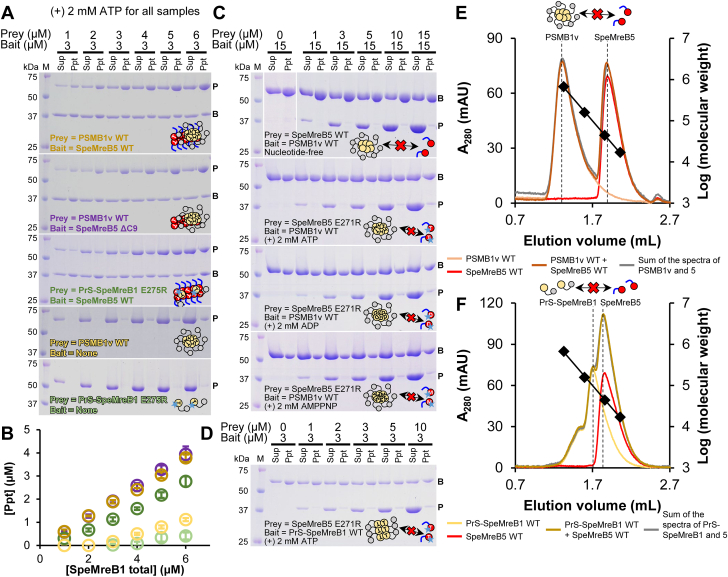
**Interaction between SpeMreB1 and SpeMreB5.** For cosedimentation assays, protein size standards were visualized in lane M with the molecular masses of each band on the *left side*. The band positions of prey and bait in the gel images are indicated by P and B, respectively. The result scheme of each experiment is shown as a *cartoon* reflecting the assembly states (monomeric, polymerized, or oligomerized), nucleotide states (empty, T, D, and M symbols for Nf, ATP, ADP, and AMPPNP, respectively), and mutations (star for the polymerization-deficient mutation and the lack of *blue line* for the C-terminal deletion of SpeMreB5) of PrS-SpeMreB1 (*yellow circle* connected with a *gray circle* indicating PrS) and SpeMreB5 (*red circle* with the C-terminal region as a *blue line*). *A,* cosedimentation assays of PrS-SpeMreB1 and SpeMreB5 variants in the pairs indicated in each gel image in the presence of 2 mM ATP. The concentration of SpeMreB5 variants was constant at 3 μM, whereas that of PrS-SpeMreB1 variants varied from 1 μM to 6 μM. *B,* quantification of pellet amounts of PrS-SpeMreB1 variants in the cosedimentation assays with the 3 μM SpeMreB5 variant. The resulting concentrations of the precipitated fractions were plotted against the total concentrations by *open circles* with the matched color scale to *A*. Error bars indicate the SD of three independent measurements. *C,* cosedimentation assays of PSMB1v WT and SpeMreB5 WT in the Nf condition or SpeMreB5 E271R in the presence of 2 mM ATP, ADP, or AMPPNP. The concentration of PSMB1v WT was constant at 15 μM, whereas that of SpeMreB5 variants varied from 0 μM to 15 μM. The centrifugation time was set to 30 min, which was short enough to minimize the sedimentation of the high concentration of SpeMreB5 monomers. *D,* cosedimentation assay of 3 μM PrS-SpeMreB1 WT and 0 μM to 10 μM SpeMreB5 E271R in the presence of 2 mM ATP. *E* and *F,* size-exclusion chromatography of (*E*) PSMB1v WT and (*F*) PrS-SpeMreB1 WT with SpeMreB5. Peak positions of PSMB1v WT, PrS-SpeMreB1 WT, and SpeMreB5 WT are indicated by *dashed lines*. Bovine thyroglobulin (670 kDa), bovine γ-globulin (158 kDa), chicken ovalbumin (44 kDa), and horse myoglobin (17 kDa) were used for the protein size standards, and their elution volumes are plotted by *closed diamonds* with the linear fit over the log of their molecular weights. *E,* gel filtration spectra of 10 μM PSMB1v WT (*light orange*), 10 μM SpeMreB5 WT (*red*), the mixture of 10 μM PSMB1v WT and 10 μM SpeMreB5 WT (*brown*), and the sum of spectra from independent loads of 10 μM PSMB1v WT and 10 μM SpeMreB5 WT (*gray*). *F,* gel filtration spectra of 10 μM PrS-SpeMreB1 WT (*yellow*), 10 μM SpeMreB5 WT (*red*), the mixture of 10 μM PrS-SpeMreB1 WT and 10 μM SpeMreB5 WT (*dark yellow*), and the sum of spectra from independent loads of 10 μM PrS-SpeMreB1 WT and 10 μM SpeMreB5 WT (*gray*). Nf, nucleotide-free.

Both PSMB1v and PrS-SpeMreB1 E275R were precipitated by ultracentrifugation in the presence of ATP-polymerized SpeMreB5 (Fig. 2, *A* and *B*), although PrS itself did not precipitate, even in the presence of SpeMreB5 and/or ATP (Fig. S4*A*). These results indicate that SpeMreB1 binds to SpeMreB5 filaments, which is probably not the artifact by the formation of PSMB1v or the E275R mutation. Deletion of the C-terminal nine SpeMreB5 residues involved in the membrane binding and nucleating the bundle formation (SpeMreB5 ΔC9 variant) (26, 28) did not affect its affinity for PSMB1v (Fig. 2, *A*, second top gel, *B*).

Next, we attempted to detect the interaction between SpeMreB5 monomers and SpeMreB1. Due to the large size of PSMB1v, it exhibited noticeable sedimentation at a high concentration. Taking advantage of this property, we performed cosedimentation assays of PSMB1v and monomeric SpeMreB5 WT under Nf conditions or SpeMreB5 E271R over the nucleotide states (Fig. 2*C*). Most SpeMreB5 molecules remained in the supernatant under all conditions. In addition, we performed cosedimentation assays with PrS-SpeMreB1 WT and SpeMreB5 E271R in the presence of ATP to study the interaction between polymerized SpeMreB1 and SpeMreB5 monomers (Fig. 2*D*). Most SpeMreB5 E271R molecules remained in the supernatant, whereas PrS-SpeMreB1 precipitated. The interaction between the SpeMreB5 monomer and SpeMreB1 variants was further assessed using size-exclusion chromatography (Fig. 2, *E* and *F*). The peak positions and magnitudes did not change in the mixed conditions of PSMB1v or PrS-SpeMreB1 and SpeMreB5 compared with when each was independently loaded. These results indicate that the polymerized form of SpeMreB5 is essential for the interaction between SpeMreB1 and SpeMreB5.

### SpeMreB1 decreases SpeMreB5 filament amount depending on the nucleotide states

**Figure 3:**
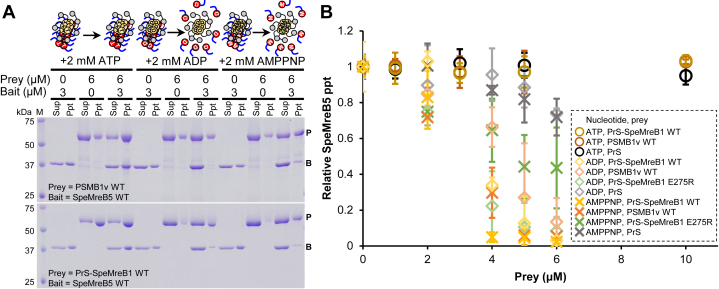
**Effects of SpeMreB1 on SpeMreB5.***A,* cosedimentation assays of 3 μM SpeMreB5 WT and 6 μM PSMB1v WT (*top*) or PrS-SpeMreB1 WT (*bottom*) in the presence of 2 mM ATP, ADP, or AMPPNP. Protein size standards were visualized in lane M with the molecular masses of each band on the *left side*. The band positions of prey and bait in the gel images are indicated by P and B, respectively. The result scheme of each cosedimentation assay of PSMB1v and SpeMreB5 is shown as a *cartoon* reflecting the assembly states (monomeric, polymerized, or oligomerized) and nucleotide states (empty, T, D, and M symbols for nucleotide-free, ATP, ADP, and AMPPNP, respectively) of PrS-SpeMreB1 (*yellow circle* connected with *gray circle* indicating PrS) and SpeMreB5 (*red circle* with the C-terminal region as a *blue line*). *B,* relative pellet amounts of SpeMreB5 WT in cosedimentation assays with an SpeMreB1 variant and a nucleotide. The total SpeMreB5 WT concentration was constant at 3 μM, whereas that of PrS-SpeMreB1 variants varied from 0 μM to 10 μM as shown in Fig. S4, *B*–*D*. The resulting concentrations of the precipitated fractions were plotted against the total concentrations of an SpeMreB1 variant. The colors of the plots indicate the proteins added *versus* SpeMreB5 WT (*yellow*-, *orange*-, *green*-, and *gray-scaled* colors for PrS-SpeMreB1 WT, PSMB1v WT, PrS-SpeMreB1 E275R, and PrS, respectively). The shapes of the plots indicate the added nucleotide (*open circles*, *open diamonds*, and *crosses* for 2 mM ATP, ADP, and AMPPNP, respectively). Error bars indicate the SD from three independent measurements.

A previous study demonstrated that SMreB1 expressed in *E. coli* cells inhibited the filament formation of SMreB2, which was coexpressed as a model of a phylogenetic group including SMreB5 (37). Inspired by this study, we evaluated the effects of SpeMreB1 on SpeMreB5 filaments using cosedimentation assays in the presence of different nucleotides (Figs. 3, *A* and *B*, S4, *B*–*D*). In the presence of ATP, the amount of SpeMreB5 precipitate did not change when mixed with approximately thrice higher concentrations of PrS-SpeMreB1 or PSMB1v. Intriguingly, in the presence of ADP or AMPPNP, the amount of SpeMreB5 precipitate decreased in the presence of PrS-SpeMreB1 or PSMB1v. In these nucleotide states, SpeMreB5 mostly did not precipitate in the presence of twice the concentration of PrS-SpeMreB1 or PSMB1v. This phenomenon also occurred with PrS-SpeMreB1 E275R in the presence of ADP (in the presence of AMPPNP and PrS-SpeMreB1 E275R, the sedimentation of SpeMreB5 did not decrease below 55%). In contrast, the precipitate decrease in SpeMreB5 by PrS under conditions corresponding to SpeMreB1 variants remained at 26.1% and 28.1% in the presence of ADP and AMPPNP, respectively. These results suggest that SpeMreB1 dissociated SpeMreB5 filaments.

### SpeMreB1 polymerization is essential for *Spiroplasma* swimming

The aforementioned experiments revealed that the polymerization-deficient variant of SpeMreB1 partially retained the ATPase activity, interaction with SpeMreB5 filaments, and the ability to decrease SpeMreB5 filaments (Figs. 1, *G* and *H*, 2, *A* and *B*, 3, *A* and *B*). To examine the significance of SpeMreB1 polymerization in *Spiroplasma* swimming, we investigated the swimming behavior of cells expressing an SpeMreB1 variant with SpeMreB5 WT using the reconstitution system of the minimal synthetic bacterium JCVI-syn3B (14). In this analysis, SpeMreB1 was untagged, as the N-terminal fusion of PrS to SpeMreB1 impaired the reconstitution of the swimming motility, despite producing cells with elongated morphology (Fig. S5 and Movie S2).

**Figure 4:**
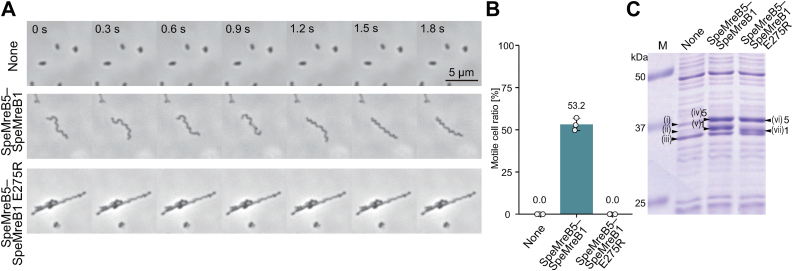
**Behavior of syn3B cells expressing an SpeMreB1 variant and SpeMreB5 WT.***A,* time-lapse phase-contrast microscopy images of representative syn3B cells taken every 0.3 s. Shown are cells carrying the antibiotic selection maker (*top*), those coexpressing SpeMreB1 WT and SpeMreB5 WT (*middle*), and SpeMreB1 E275R and SpeMreB5 WT (*bottom*). Time 0 indicates the start of image acquisition. Scale bar represents 5 μm. *B,* the motile cell ratio of the syn3B-expressing SpeMreBs analyzed for three individual fields (110.8 × 62.3 μm^2^) including 911, 545, and 563 cells, respectively. Each data point is plotted as an *open circle* on the chart. *C,* protein profiles of syn3B strains. Protein size standards are shown in *lane M* with the molecular masses of each band on the *left side*. The marked protein bands were identified by peptide mass fingerprinting as summarized in Table S1. The bands of SpeMreB1 and SpeMreB5 are marked as 1 and 5, respectively.

Although syn3B cells coexpressing SpeMreB1 WT and SpeMreB5 WT were motile, as observed in *Spiroplasma*, those coexpressing SpeMreB1 E275R and SpeMreB5 WT were not (Fig. 4, *A* and *B* and Movie S1). Protein profile analysis revealed that bands corresponding to the SpeMreB1 variant and SpeMreB5 WT were detected in both strains (Fig. 4*C*, Table S1), suggesting that the loss of swimming motility in the cells coexpressing SpeMreB1 E275R and SpeMreB5 WT was not caused by aberrant SpeMreB expression. These results indicate that SpeMreB1 polymerization is essential for *Spiroplasma* swimming.

### SpeMreB1 binds to negatively charged lipids in any nucleotide state

**Figure 5:**
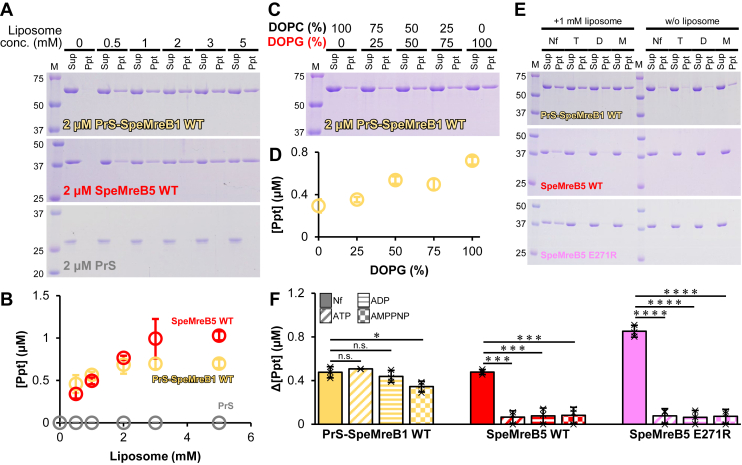
**Liposome binding of SpeMreBs.** For gel images, protein size standards were visualized in lane M with the molecular masses of each band on the *left side*. *A,* liposome binding assays of 2 μM PrS-SpeMreB1 WT (*top*), 2 μM SpeMreB5 WT (*middle*), and 2 μM PrS (*bottom*) for concentrations of SpiroLipid liposomes. *B,* quantification of pellet amounts in the liposome binding assays. The resulting concentrations of the precipitated fractions were plotted over the total liposome concentrations by *yellow* (2 μM PrS-SpeMreB1 WT), *red* (2 μM SpeMreB5 WT), and *gray* (2 μM PrS) *open circles*. Error bars indicate the SD of three independent measurements. *C,* liposome binding assay of 2 μM PrS-SpeMreB1 WT in the presence of 1 mM liposomes with different DOPC and DOPG ratios shown at the top of each lane. DOPG is labeled in *red characters* as indicative of its negative charge. *D,* quantification of pellet amounts in the liposome binding assays of 2 μM PrS-SpeMreB1 WT in the presence of 1 mM liposomes with different DOPC and DOPG ratios plotted against the DOPG ratio. Error bars indicate the SD of three independent measurements. *E,* liposome binding assays of 2 μM PrS-SpeMreB1 WT (*top*), 2 μM SpeMreB5 WT (*middle*), and 2 μM SpeMreB5 E271R (*bottom*) in the presence of 1 mM SpiroLipid liposome and 1 mM nucleotide as indicated at the top of each lane (Nf, T, D, M for nucleotide-free, with ATP, with ADP, and with AMPPNP, respectively). *F,* quantification of pellet amounts in the liposome binding assays in the presence of 1 mM SpiroLipid liposome and 1 mM nucleotide. Precipitate concentration differences in the presence and absence of liposomes were summarized for each construct. The patterns in the bars indicate the added nucleotides (*solid*, *diagonal stripe*, *horizontal stripe*, and *check patterns* for nucleotide-free, with ATP, ADP, and AMPPNP, respectively). Error bars indicate the SD of three independent measurements. Each data point is plotted as a cross on the chart. Symbols indicate *p* values supported by Student’s *t* test (∗*p* < 0.05, ∗∗∗*p* < 1.0 × 10^−3^, ∗∗∗∗*p* < 1.0 × 10^−4^, and n.s. *p* > 0.05).

As the motility machinery of *Spiroplasma* is present underneath the cell membrane (13, 38, 39, 40), it is important to study the membrane-binding ability of each component of the motility machinery. Although the membrane binding of SMreB1 is largely unclear, the binding ability of SMreB5 to negatively charged lipids has been experimentally demonstrated (28). Therefore, we investigated the membrane binding of PrS-SpeMreB1 based on the cosedimentation assay with liposomes. In this assay, we reduced the centrifugation speed (from 436,000*g* to 100,000*g*) and time (from 90 to 30 min) in the sedimentation assays without liposomes to reduce the background sedimentation of SpeMreB filaments. PrS-SpeMreB1 monomers coprecipitated with liposomes with a lipid composition mimicking that of the *Spiroplasma* cell membrane (SpiroLipid liposome), whereas PrS did not (Fig. 5*A*), indicating that SpeMreB1 binds to the membrane, contrary to our previous prediction based on the absence of membrane-binding sequences common to MreB family proteins (15). The precipitated fractions of PrS-SpeMreB1 and SpeMreB5 were 34.9% and 51.4%, respectively (Fig. 5*B*), at the saturated liposome concentration, indicating that the membrane-binding ability of SpeMreB1 is slightly weaker than that of SpeMreB5.

To identify the lipid molecules involved in the binding to SpeMreB1, we performed liposome-binding assays of PrS-SpeMreB1 using liposomes composed of one of the following four lipids in the SpiroLipid liposome: 1,2-dioleoyl-*sn*-glycero-3-phospho-(1′-rac-glycerol) (DOPG), 1,2-dioleoyl-*sn*-glycero-3-phosphocholine (DOPC), sphingomyelin (SM), and cardiolipin (CL) (Fig. S4*E*). PrS-SpeMreB1 bound to all four liposomes but strongly bound to those composed of DOPG or CL, both of which are negatively charged. To further examine the negative charge dependence of lipids for binding with SpeMreB1, we performed liposome-binding assays of PrS-SpeMreB1 using liposomes with different ratios of DOPG and DOPC. As the ratio of DOPG increased, the affinity between PrS-SpeMreB1 and the liposomes became high (Fig. 5, *C* and *D*). These results indicate that SpeMreB1 binds to the membrane including negatively charged lipids like SMreB5 (28). To confirm the consistency of the negatively charged lipid dependence of the membrane binding of SpeMreB1, we modeled the structure of full-length SpeMreB1 using AlphaFold2 (41) (Fig. S4*F*). MreB is composed of four subdomains (IA, IB, IIA, and IIB) (16, 20, 27, 28, 32, 34), in which the side of the subdomains IA and IB is involved in the membrane binding of all studied MreBs (21, 28). SpeMreB1 possesses a positively charged region throughout its IA and IB subdomains, allowing it to bind to the membrane.

Next, we evaluated the membrane binding of SpeMreB1 to liposomes in the presence of nucleotides (Fig. 5, *E* and *F*). Even in the presence of nucleotides, PrS-SpeMreB1 and SpeMreB5 precipitate poorly at a reduced centrifugation force in the absence of liposomes. SpeMreB5 did not bind to the liposomes in the presence of nucleotides, which is consistent with a previous study (28). This phenomenon was independent of polymerization, as was confirmed by SpeMreB5 E271R. In contrast, the cosedimentation amounts of PrS-SpeMreB1 with liposomes were less affected than that of SpeMreB5. These results indicate that the membrane-binding region of SpeMreB1 is still functional after binding to a nucleotide, unlike SpeMreB5.

## Discussion

**Figure 6:**
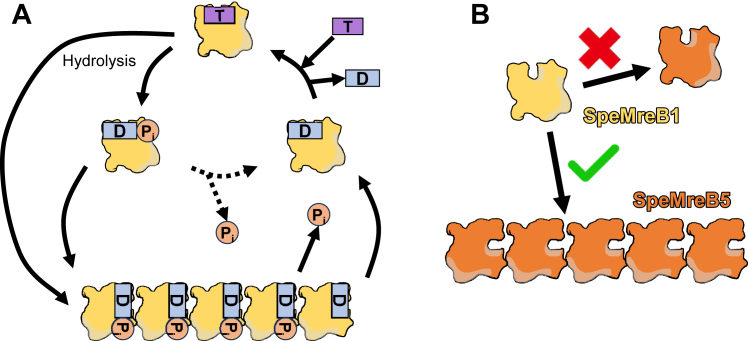
**Working models and summary of SpeMreB1 molecular features.***A,* working model of the polymerization dynamics of SpeMreB1. The monomeric SpeMreB1 hydrolyzes ATP with a probable rate-limiting step of P_i_ release. During this cycle, the monomer in the ATP and/or ADP-P_i_ state enters the polymerization cycle, resulting in a higher P_i_ release rate. *B,* mode of SpeMreB1 binding to SpeMreB5 filaments but not to SpeMreB5 monomers.

In this study, we solubilized SpeMreB1 by fusing it with PrS and evaluated its activity, crosstalk with SpeMreB5, and membrane binding. Simultaneously with nucleotide-dependent polymerization (Fig. 1, *C*–*F*), we found the highest fold changes in C_C_ in nucleotide states and P_i_ release rates of PrS-SpeMreB1 in the MreB family proteins (27, 28, 32, 33, 35, 36, 42, 43, 44, 45) (Tables 1 and 2). These findings suggest that SpeMreB1 is exceptionally dynamic in the MreB family proteins. The fusion of PrS may have reduced the intrinsic SpeMreB1 activity by possibly decreasing the diffusion rate of SpeMreB1 monomers and creating steric hindrance, resulting in slow polymerization and failure to reconstitute the swimming motility in syn3B cells (Fig. S5 and Movie S2). However, these possible effects do not alter the aforementioned conclusion. A previous study demonstrated that SMreB1 filaments formed in *E. coli* cells were static (37). Our finding of SpeMreB1 bundles (Fig. 1*D*) suggests that the filaments observed in *E. coli* cells were in the form of bundles. Based on experiments using polymerization-deficient variants (Fig. 1, *G*–*I*), we confirmed the ATPase futile cycle in PrS-SpeMreB1 and SpeMreB5, as monitored in GsMreB (32). Time-course P_i_ release measurements of PrS-SpeMreB1 E275R and SpeMreB5 E271R revealed that the P_i_ concentration increased proportionally with time (Fig. 1*G*). Based on these findings, we proposed an updated SpeMreB polymerization cycle (Fig. 6*A*). First, SpeMreBs can hydrolyze ATP even in the monomeric state, with P_i_ release likely being the rate-limiting step. Monomeric SpeMreBs in either ATP or ADP-P_i_ states are most favorable for initiating polymerization, leading to accelerated P_i_ release. In the case of SpeMreB1, most monomers probably polymerize in the ADP-P_i_ state, given their low polymerization ability in the presence of AMPPNP (Fig. 1*E*). In contrast, SpeMreB5 displayed only twofold differences in C_C_ in the presence of AMPPNP and ATP (27) (Table 1) and slow P_i_ release in the polymerization-deficient variant (Table 2, Fig. 1*H*), suggesting that SpeMreB5 can polymerize before hydrolyzing ATP. In this reaction path, SpeMreB5 follows a polymerization cycle without considering the ATPase cycle in the monomeric state, as previously described (27).

Polymerization of SpeMreB5 is essential for the interaction between SpeMreB1 and SpeMreB5 (Figs. 2 and 6*B*). In addition, we found a decrease in SpeMreB5 filaments in the presence of SpeMreB1, depending on the nucleotide state (Fig. 3, *A* and *B*). This result is consistent with a previous study, in which the filament formation of SMreB2, the most identical SMreB to SMreB5, in *E. coli* cells was inhibited by the coexpression of SMreB1 (37). This phenomenon was probably not caused by either the sequestration of SpeMreB5 monomers by SpeMreB1 (Fig. 3*B*) or by copolymerization of SpeMreB1 and SpeMreB5 (Fig. 2, *A* and *B*). Assuming that MreB5 filament destabilization is involved in the force generation cycle of *Spiroplasma* swimming, it is plausible that SpeMreB1, rather than inhibiting polymerization, destabilizes SpeMreB5 filaments, which may be essential to prevent the polymerization dynamics from stalling. Polymerization of SpeMreB1 was essential for *Spiroplasma* swimming (Fig. 4), suggesting that the interaction between polymerized SpeMreB1 and SpeMreB5 is essential in force generation for this motility. Although the decrease in SpeMreB5 filaments by SpeMreB1 depended on their nucleotide states, the atomic structures of SMreB5 were not substantially different between the ADP- and AMPPNP-bound states (28). Therefore, the decrease in the SpeMreB5 filament amount may be due to the differences of its mechanical properties across the nucleotide state. A previous study has reported that actin filaments become flexible upon P_i_ release, whereas their structure remains largely unchanged. The change in the mechanical properties of actin filaments has been thought to affect the specificity of actin-binding proteins, such as cofilin, which selectively severs actin filaments in the ADP-bound state (46).

We found that SpeMreB1 bound to a membrane containing negatively charged lipids (Fig. 5). In walled bacteria, MreB binds to the membrane *via* an amphipathic helix at the N terminus and/or consecutive hydrophobic residues in the hydrophobic loop region in subdomain IA (21). Because most SMreB1s and SMreB4s do not possess these sequences, we predicted that SMreB1 and SMreB4 would not bind to the membrane (15). However, SpeMreB1 bound to the membrane even without the membrane-binding regions common to walled-bacterial MreB (Fig. 5). This finding suggests that SpeMreB1 functions underneath the cell membrane. In addition, SMreB5 binds to the membrane *via* its positively charged C-terminal region, which has not been reported in the MreBs of walled bacteria (28). These findings suggest that SMreB1 and SMreB5 have evolved unique membrane-binding mechanisms for driving *Spiroplasma* swimming. In this study, we confirmed the dissociation of SMreB5 from the membrane in the presence of a nucleotide (Fig. 5, *E* and *F*), although independently expressed SMreB1 and SMreB5 in *Mycoplasma mycoides* localized underneath the cell membrane as filaments (13). This inconsistency suggests that other factors that are not reproducible in *in vitro* liposome-binding assays, such as membrane curvature and the excluded volume effect, may control the membrane binding of SMreB filaments.

In conclusion, we demonstrated the dynamic properties, membrane-binding ability, and crosstalk with SpeMreB5 filaments of SpeMreB1. These findings suggest that SpeMreB1 functions as a “molecular motor” to exert the force on a “cytoskeleton” composed of relatively static SpeMreB5 filaments to propel *Spiroplasma* swimming.

## Experimental procedures

### Phylogenetic analysis

The phylogenetic tree (Fig. S1*B*) was constructed using the maximum likelihood method based on all SMreB1 and SMreB4 sequences reported until March 3, 2021, as previously described (15). Bootstrap supports were estimated from 1000 alignment samples. Ancestor estimation was performed using MEGA-X (downloaded from https://www.megasoftware.net/), as described previously (15, 47).

### Cloning and expression of SpeMreB

Constructs for the expression of SpeMreB1, SpeMreB4, and SpeMreB5 fused with a 6× His-tag were prepared as the fusions of these genes with codon optimization for *E. coli* expression and a pCold-15b vector, as described in our previous study (27). Expression plasmids for the other SMreB1s and SMreB4s were constructed as fusions of pCold-15b as described previously (27). The plasmid for coexpressing SpeMreB4 and SpeMreB5 was constructed by inserting the codon-optimized *spemreB5* gene with the 5′-extension of GGATCCTAATTTTGTTTAACTTTAAGAAGGAGATAT, which carries an *E. coli* ribosome-binding site to the downstream of the codon-optimized *spemreB4* gene in pCold-15b. To construct expression plasmids for PrS-SpeMreB1 and PrS-SpeMreB4, the codon-optimized genes for *E. coli* expression were excised using NdeI and BamHI restriction enzymes and inserted into pCold-PrS (provided by Dr Yoshihiro Yamaguchi, Osaka Metropolitan University, Japan), in which two consecutive PrS molecules are fused to the N terminus of the protein of interest (Data S1) and expressed by the pCold expression system with the selective marker of ampicillin. These SpeMreBs carry the N- and C-terminal extensions MNHKVHHHHHHMANITVFYNEDFQGKQVDLPPGNYTRAQLAALGIENNTISSVKVPPGVKAILYQNDGFAGDQIEVVANAEELGPLNNNVSSIRVISVPVQPRMANITVFYNEDFQGKQVDLPPGNYTRAQLAALGIENNTISSVKVPPGVKAILYQNDGFAGDQIEVVANAEELGPLNNNVSSIRVISVPVQPRGTIEGRH and GSRGEIHHHHHH, respectively. An empty pCold-PrS vector was used to express PrS. *Thermotoga maritima* MreB (TmMreB) was constructed as a fusion with an 8× His-tag by inserting the codon-optimized sequence to pSY5 vector (48). Each construct was transformed into *E. coli* BL21 (DE3) cells. The *E. coli* strains were grown overnight in the LB medium in the presence of 50 μg/ml ampicillin at 37 °C. The overnight culture was diluted with fresh LB medium containing 50 μg/ml ampicillin and incubated at 37 °C. Protein expression was induced at the growth point with an absorbance at 600 nm of 0.4 to 0.6 in the presence of 1 mM IPTG for 24 h at 15 °C. Cells were harvested, washed twice with PBS (10 mM Na_2_HPO_4_, 2 mM NaH_2_PO_4_, 3 mM KCl, and 137 mM NaCl), and stored at −80 °C until needed.

### Purification of SpeMreB

SpeMreB1, SpeMreB4, SpeMreB5, TmMreB, and their variants were purified by Ni^2+^–NTA affinity chromatography and gel filtration as previously described (27, 33, 49) independent of fusion with PrS. Briefly, cell pellets were resuspended in His trap buffer A (50 mM Tris–HCl [pH 8.0], 300 mM NaCl, 50 mM imidazole–HCl [pH 8.0]), centrifuged at 12,000*g* for 30 min at 4 °C, and purified using HisTrap HP 5 ml (Cytiva). The imidazole concentration in the elution buffer for SpeMreB1 was changed from that used for SpeMreB5 (230 mM) to 500 mM because of the presence of double 6× His tags. The purified SpeMreBs were further subjected to HiLoad 26/600 Superdex 200 pg (Cytiva) in the gel filtration buffer (20 mM Tris–HCl [pH 8.0], 300 mM NaCl) at 4 °C. PrS was purified by Ni^2+^–NTA affinity chromatography using the same procedure as that used for SpeMreBs. Protein concentrations were determined from the absorbance at 280 nm measured using a NanoDrop One (Thermo Fisher Scientific), with the absorption coefficients estimated from ProtParam, as previously described (49).

### Electron microscopy

A 4 μl sample drop was placed onto a 400-mesh copper grid coated with carbon for 1 min at room temperature (23–27 °C), washed with 10 μl water, stained with 2% (w/v) uranyl acetate for 45 s, air dried, and observed under a JEOL JEM-1010 transmission electron microscope at 80 kV equipped with a FastScan-F214T charge-coupled device camera (TVIPS). The image averaging of PrS-SpeMreB1 sheet was performed by CryoSPARC, version 4.6.0 (50) with manually selected 1284 particles. The major axis of PSMB1v was estimated by the distance measurement tool of ImageJ (National Institutes of Health; http://rsb.info.nih.gov/ij/).

### Sedimentation assays

Samples were prepared in buffer S (20 mM Tris–HCl [pH 8.0], 1 M NaCl, 200 mM l-arginine–HCl [pH 8.0], 5 mM DTT, 2 mM MgCl_2_, and 2 mM of the desired nucleotide), which was used in a previous study to evaluate the polymerization activities of SpeMreB3 and SpeMreB5 (27). The reliability of the buffer S was confirmed by the sedimentation assay of TmMreB, where the band pattern was consistent with a previous sedimentation assay using KMEI buffer, which is commonly used for actin polymerization (Fig. S2*H*) (43). Cosedimentation assays containing two of SpeMreB1, SpeMreB5, and PrS in the same sample solution were performed using this method. Sample incubation was initiated by mixing the sample solution with the desired protein concentration in gel filtration buffer and a reaction premix containing the other reagents in buffer S. After incubation at room temperature for 3 h, which was long enough to reach the equilibrium state of the polymerizations as confirmed by SpeMreB3 and SpeMreB5 (27), the 200 μl samples were subjected to ultracentrifugation at 100,000 rpm at 23 °C using a TLA-100 rotor (Beckman Coulter). The centrifugation time was set to 90 min (for samples containing PrS-SpeMreB1 variants) or 120 min (for samples without PrS-SpeMreB1), which is short enough to minimize the sedimentation of SpeMreB monomers unless otherwise stated. After removal of the supernatant, the pellet was resuspended in 200 μl of water. The supernatant and pellet fractions were analyzed by SDS-PAGE on a 12.5% Laemmli gel and stained with Coomassie Brilliant Blue R-250. Of note, when preparing PrS-SpeMreB1-containing samples for SDS-PAGE, we omitted the steps of heat shock (at 95 °C for 3 min in our laboratory protocol) or overnight incubation (at room temperature for more than 7 h in our laboratory protocol) after mixing with an SDS-PAGE sample solution (5% [v/v] glycerol, 0.025% [w/v] bromophenol blue, 62.5 mM Tris–HCl [pH 6.8], 2.5% [w/v] sodium dodecyl sulfate, 5% [v/v] β-mercaptoethanol in our laboratory recipe), which are widely used for protein denaturation (51), because we have the following two experiences; (I) these steps stimulate the aggregation of PrS-SpeMreB1 in the SDS-PAGE sample solution and smearing of PrS-SpeMreB1 bands (Fig. S2*I*) and (II) all proteins used in this study were denatured immediately after mixing with the SDS-PAGE sample solution, as confirmed by the small amounts of smeared bands (see gel images in this paper, except Fig. S2*I*, where the SDS-PAGE samples were prepared by the 95 °C heat shock). The concentrations of the supernatant and pellet fractions were estimated as the proportion of the total SpeMreB concentration and the ratio of each fraction to the sum of the supernatant and pellet fractions measured with the ImageJ. The C_C_ was estimated as the *x*-intercept of the linear fit with the amount of sedimentation at steady-state polymerization *versus* the total SpeMreB concentration.

### P_i_ release assays

P_i_ release measurements were performed as previously described in a standard buffer for SpeMreB polymerization (20 mM Tris–HCl [pH 7.5], 100 mM KCl, 5 mM DTT, 2 mM MgCl_2_, and 2 mM ATP) (27, 49). Briefly, P_i_ release was detected using 2-amino-6-mercapto-7-methylpurine riboside, a molecular probe for P_i_ that reacts with P_i_ to produce a molecule that absorbs light at 360 nm.

### Circular dichroism

CD was measured for a 200 μl sample in a 1 mm thick quartz cuvette by J720W (JASCO) with a wavelength range of 200 to 250 nm under a nitrogen gas flow of 6 l/min at 20 °C maintained by a temperature stabilizer. The measured CD intensities were converted to the mean residue ellipticities (MREs) using the following equation:

(1) $$\text{MRE}\;\left(\deg\;{\text{cm}}^{2}\;{\text{dmol}}^{-1}\right)=\frac{\text{CD}\;\left(\text{mdeg}\right)}{N\cdot C\;\left(M\right)\cdot l\;\left(\text{mm}\right)}$$

where *N* is the number of amino acid residues in the protein of interest, *C* is the protein concentration (*M*), and *l* is the path length of the cuvette used for measurements (mm). Secondary structure contents were estimated using BeStSeL (52, 53, 54) and compared with those estimated from the amino acid sequences using PSIPRED4 (55).

### Size-exclusion chromatography to study the interactions between SpeMreB1 and SpeMreB5

The samples were mixed in 20 mM Tris–HCl (pH 7.5), 200 mM KCl, and incubated on ice for 1 h. A 100 μl sample solution was loaded onto Superdex 200 Increase 3.2/300 (Cytiva) equilibrated by 20 mM Tris–HCl (pH 7.5), 200 mM KCl at 4 °C. Protein signals were detected by measuring the absorbance at 280 nm.

### Protein expression in JCVI-syn3B

The construct for transferring *spemreB1* and *spemreB5* into the genome of *M. mycoides* JCVI-syn3B (GenBank: CP069345.1) was assembled using pSD079 (56) and three DNA fragments amplified from the *S. eriocheiris* genome (GenBank: GCA_002028345.1) (57): 496,781st to 498,064th nucleotides, including *spemreB1* and its promoter*;* 1,353,199th to 1,353,517th, including the promoter of *spemreB4*; and 1,354,572nd to 1,355,801st, including *spemreB5* and its terminator. The construct for *spemreB1* E275R and *spemreB5* was prepared by inducing a mutation in pSD079 carrying *spemreB1* WT and *spemreB5* WT. The construct for *PrS-spemreB1* and *spemreB5* was prepared by transferring the gene for N-terminally His-tagged PrS expression with the optimized codons for syn3B to the 5′-end of *spemreB1* WT for expression of PrS-SpeMreB1 without the C-terminal 6× His-tag. pSD128 was used as a control strain carrying the puromycin resistance gene *puroR*. Each construct was transferred into JCVI-syn3B (58, 59) as previously described (14). These strains were cultured in the SP4 medium at 37 °C to 0.03 to 0.04 at an absorbance at 620 nm. For protein profiling, the cell suspensions were concentrated 20-fold after washing with PBS containing sucrose (PBS/S) (75 mM sodium phosphate [pH 7.3], 68 mM NaCl, and 20 mM sucrose) and analyzed by SDS-PAGE. Protein bands were identified as previously described (38).

### Optical microscopy and video analyses of JCVI-syn3B cells

The cultures were centrifuged at 9000*g* for 8 min at 10 °C. The cell pellets were resuspended in PBS/S at one-fifth the volume of the culture, mixed with methylcellulose in PBS/S to a final concentration of 0.2%, and loaded into the tunnel slides. After 5 min at 25 °C, 0.4% methylcellulose in PBS/S was inserted into the tunnel slide to wash out floating cells, and cells remaining near the glass surface were observed using an inverted microscope IX71 (Olympus) equipped with a UPlanSApo 100× 1.4 numerical aperture Ph3 and complementary metal-oxide-semiconductor camera, DMK33UX174 (The Imaging Source Asia Co, Ltd). The video was analyzed using the empirical gradient threshold plugin (60) and a color footprinting macro (61) of ImageJ, version 1.53f51 (Fiji) (62).

### Liposome preparation

Chloroform solutions of 10 mg/ml DOPG (840475C; Avanti Polar Lipid), DOPC (850375C; Avanti Polar Lipid), SM (860062C; Avanti Polar Lipid), and CL (710335C; Avanti Polar Lipid) were used for liposome construction. For SpiroLipid liposomes, chloroform solutions of these lipids were mixed in a previously described molar ratio (16, 63) (DOPG:DOPC:SM:CL = 0.38:0.14:0.33:0.15). For liposome preparation, a chloroform solution with the desired lipid composition was aliquoted into a clean glass bottle and was completely dried by N_2_ gas flow and a vacuum desiccation at room temperature. The remaining lipid layer was hydrated at room temperature using a standard buffer with increasing KCl concentration to 200 mM (to reduce the background sedimentation of SpeMreBs) containing 1 mM MgCl_2_ (for efficient liposome construction) and subjected to water bath sonication for 1 min. The liposome solution was extruded at room temperature using a 100 nm polycarbonate membrane (610005; Avanti Polar Lipid). The liposome solution was stored at 4 °C and used within 2 days from the construction.

### Liposome binding assays

SpeMreB samples were dialyzed into 20 mM Tris–HCl (pH 7.5), 200 mM KCl, 1 mM MgCl_2_ at 4 °C for overnight. The dialyzed samples were centrifuged at 20,000*g* for 10 min at 4 °C to remove aggregates, mixed with a liposome solution with a final SpeMreB concentration and sample volume of 2 μM and 200 μl, respectively, and incubated for 15 min at room temperature. For liposome binding assays in the presence of a nucleotide, SpeMreBs were incubated in the presence of a nucleotide for 5 min at room temperature before liposome addition at twice the SpeMreB and nucleotide concentrations (4 μM and 2 mM, respectively). The incubated samples were centrifuged at 48,000 rpm (approximately 100,000*g*) for 30 min at 23 °C using a TLA-100 rotor. The supernatant was removed, and the pellet was resuspended in water. SpeMreB concentrations in each fraction were estimated by SDS-PAGE and subsequent image analysis as described in “Sedimentation assays” in the Experimental procedures section.

## Data availability

Raw data are available from the corresponding author upon reasonable request.

## Supporting information

This article contains supporting information (21, 28, 41).

## Conflict of interest

The authors declare that they have no conflicts of interest with the contents of this article.
