# Supplementary material for: A bacterial actin with high ATPase activity regulates the polymerization of a partner MreB isoform essential for Spiroplasma swimming motility
Source: J Biol Chem. 2025 Jul 7;301(8):110462. doi: 10.1016/j.jbc.2025.110462 (PMC12337190; doi:10.1016/j.jbc.2025.110462)
Supplement: Supporting information [file mmc1.pdf]

Supporting information for

A highly active bacterial actin actuates the polymerization of another isoform essential for swimming motility of *Spiroplasma*

Authors

Daichi Takahashi[1,†], Hana Kiyama[1], Hideaki T. Matsubayashi[2], Ikuko Fujiwara[1,3,*], Makoto Miyata[1,4]

Affiliations

[1]Graduate School of Science, Osaka Metropolitan University, Osaka, Japan
[2]Frontier Research Institute for Interdisciplinary Sciences, Tohoku University, Miyagi, Japan
[3]Department of Materials Science and Bioengineering, Nagaoka University of Technology, Nagaoka, Niigata, Japan
[4]The OMU Advanced Research Center for Natural Science and Technology, Osaka Metropolitan University, Osaka, Japan
[*]Corresponding author: ikukofujiwara@vos.nagaokaut.ac.jp
[†]Current affiliation: Research Institute for Interdisciplinary Science, Okayama University, Okayama, Japan

Table S1. Results of peptide mass fingerprinting of bands shown in Fig. 4C.[a]

Protein band	Protein ID	Gene name	Annotation	Mascot protein score	Mass [kDa]	Sequence coverage [%]	Species
i	QWN46562.1	*gap*	Glyceraldehyde-3-phosphate dehydrogenase	39	37.2	24	JCVI-Syn3B
ii	QWN46586.1	*rpoA*	DNA-directed RNA polymerase subunit alpha	60	35.0	45	JCVI-Syn3B
iii	QWN46506.1*	*ldh*	L-lactate dehydrogenase	20	34.8	26	JCVI-Syn3B
iv	AHF58343.1	*mreB5*	Cell shape-determining protein MreB	69	38.7	28	*S. eriocheiris*
v	AHF57598.1	*mreB1*	Cell shape-determining protein MreB	53	38.0	34	*S. eriocheiris*
vi	AHF58343.1	*mreB5*	Cell shape-determining protein MreB	51	38.7	26	*S. eriocheiris*
vii	AHF57598.1-2	*mreB1 E275R*	Cell shape-determining protein MreB	59	38.0	30	*S. eriocheiris*

[a.] Roman numbers indicate protein bands shown in Fig. 4C. All proteins were detected in both peptide mass fingerprinting and MS/MS analyses. The Mascot protein score is defined by −10×log(P), where P is the probability that the observed match is a random event. The reference dataset for peptide mass finger printing was prepared by adding the amino acid sequence of SpeMreB1 E275R to the coding sequence libraries of Synthetic bacterium JCVI-syn3B (taxonomy ID: 2806337) and *Spiroplasma eriocheiris* (taxonomy ID: 743698) in the NCBI database. The protein with star was identified in the MS/MS analysis, although its score in the peptide mass finger printing.

Fig. S1. Preparation and evaluation of PrS-SpeMreB1 and PrS-SpeMreB4. For gel images, protein size standards were visualized in lane M with the molecular masses next to each band. (**A**) Expression of SpeMreB1 WT (left) and SpeMreB4 WT (right) without the PrS tag. The SpeMreBs were expressed by *E. coli* BL21 (DE3) as a 6×His-tag fusion at each N-terminus using the pCold-15b expression system. Cells were sonicated in 50 mM Tris-HCl pH 8.0, 300 mM NaCl, and centrifuged at 12,000 ×*g* for 30 min at 4°C. The whole cell lysate, supernatant, and pellet fractions were analyzed by SDS-PAGE. The band positions for SpeMreB1 WT and SpeMreB4 WT are indicated by an arrowhead. (**B**) Phylogenetic tree of SMreB1 and SMreB4. The scale bar is in units of the number of amino acid substitutions per site. Sequences used for expression experiments in this study are indicated by red triangles with their labeling. (**C**) Expression of *S. melliferum* MreB4 (SmerMreB4), *S. kunkelii* MreB4 (SkuMreB4), *S. melliferum* MreB1 (SmerMreB1), *S. apis* MreB4 (SapiMreB4), *S. litorale* MreB1 (SlitMreB1), a reconstituted ancestor of the Citri clade MreB1 and MreB4 (AnCitMreB1/4), and a reconstituted ancestor of *Spiroplasma* MreB1 and MreB4 (AnSMreB1/4). These constructs were cloned, expressed, isolated, and analyzed by SDS-PAGE using the same procedures as those in Fig. S1A. The band positions of these constructs are indicated by an arrowhead. (**D**) Co-expression of 6×His-tagged SpeMreB4 WT and untagged SpeMreB5 WT and their purification. *E. coli* cells co-expressing these SpeMreB4 and SpeMreB5 constructs were sonicated in 50 mM Tris-HCl pH 8.0, 50 mM Imidazole-HCl pH 8.0, 300 mM NaCl, centrifuged at 12,000 ×*g* for 30 min at 4°C and applied to a Ni^{2+}-NTA affinity chromatography column. The following fractions were visualized by a Coomassie-stained 12.5% Laemmli gel; fraction 1: lysate of *E. coli* co-expressing SpeMreB4 WT and SpeMreB5 WT; fractions 2 and 3: soluble (2) and insoluble (3) fractions of the whole cell lysate (1); fraction 4: flow-through fraction of Ni^{2+}-NTA affinity chromatography; other fractions: elution fractions of Ni^{2+}-NTA affinity chromatography with a gradually increasing imidazole concentration. Red and black stars indicate the

bands in which SpeMreB4 and SpeMreB5 were detected, respectively, by the peptide mass fingerprinting technique using MALDI-TOF MASS spectrometry. SpeMreB5 was detected in lanes 2, 3, and 4, whereas SpeMreB4 was detected in lane 3 and not detected in lane 2, indicating that SpeMreB4 remained insoluble despite co-expression with SpeMreB5. (**E**) Purification procedure of PrS-SpeMreB1 WT. The following fractions were visualized using a Coomassie-stained 12.5% Laemmli gel; fraction 1: lysate of *E. coli* expressing PrS-SpeMreB1 WT; fractions 2 and 3: soluble (2) and insoluble (3) fractions of the whole cell lysate (1); fractions 4 and 5: flow-through (4) and elution (5) fractions of Ni^{2+}-NTA affinity chromatography; the other fractions: gel filtration elution fractions with the elution volume in Fig. 1B indicated on each lane. The band position of PrS-SpeMreB1 is indicated by an arrowhead. (**F**) Removal of the PrS tag from PrS-SpeMreB1 WT. PrS-SpeMreB1 WT at 10 μM concentration was dialyzed against 20 mM Tris-HCl pH 7.5, 100 mM KCl (Stand.) or 20 mM Tris-HCl pH 8.0, 300 mM NaCl (Gel. fil.) for 20 h at 4°C in the presence (+) or absence (−) of 0.1 mg / ml factorXa (Novagen, Merck Co. Ltd.) and centrifuged at 20,000 ×*g* for 10 min at 4°C. The resulting supernatant and pellet fractions were visualized by SDS-PAGE. The band positions of PrS-SpeMreB1 WT, SpeMreB1 WT, factorXa, and PrS are indicated by closed triangles. The other bands are probably due to non-specific cleavage of PrS-SpeMreB1 by factorXa. (**G**) Sedimentation assays of PrS-SpeMreB1 E275R (top) and SpeMreB5 E271R (bottom) in the presence of 2 mM ATP. (**H**) Sedimentation assays of 5 μM SpeMreB4 WT in the presence or absence of 2 mM ATP.

Fig. S2. Evaluation of PSMB1v. For gel images, protein size standards were visualized in lane M with the molecular masses next to each band. (**A**) Gel filtration profiles of 50 μM PrS by Superdex™ 200 Increase 3.2/300 in the gel filtration buffer. Bovine thyroglobulin (670 kDa), bovine γ-globulin (158 kDa), chicken ovalbumin (44 kDa), and horse myoglobin (17 kDa) were used as the protein size standards, and their elution volumes are plotted using closed diamonds with the linear fit over the log of their molecular weights. The estimated elution volume of the monomeric PrS (23 kDa) shown as a dashed line is in good agreement with the peak top position of the PrS elution pattern, indicating that most of PrS molecules were monomers at this concentration. (**B–D**) Negative staining EM images of 300 nM PSMB1v in the standard buffer (**B**) without an incubation and (**C–D**) with an incubation at RT for 1 h in the (**C**) presence and (**D**) absence of 2 mM MgATP. (**E**) The size distribution of PSMB1v at the conditions shown in Fig. S2B–D. The major axes of the all-detectable particles were measured for each micrograph and accumulated until approximately 200 data were collected. Sample numbers of each condition are indicated below each box. Symbols indicate *p*-value supported by Student's *t*-test (n.s. $p > 0.05$). (**F**) Sedimentation assays of 3 μM PSMB1v WT in the presence or absence of 2 mM ATP. (**G**) CD spectra of 5 μM PrS-SpeMreB1 WT (yellow), 5 μM PSMB1v WT (orange), and 5 μM PrS (gray) in the gel filtration buffer. (**H**) Sedimentation assays of 3 μM TmMreB in the presence or absence of 2 mM ATP. (**I**) An example of an SDS-PAGE profile of PrS-SpeMreB1 WT in which the samples were prepared with a heat shock step. After mixing with the SDS-PAGE sample solution, PrS-SpeMreB1 in the gel filtration buffer was heated at 95°C for 3 min before loading onto the SDS-PAGE gel, and smears of PrS-SpeMreB1 bands appeared at the region indicated by half brackets. Black stars indicate the lanes in which multiple amounts of PrS-SpeMreB1 WT were stacked on top of stacking and running gels. The band position for unaggregated PrS-SpeMreB1 WT is indicated by an arrowhead.

Fig. S3. Negative staining EM images of PrS-SpeMreB1 and PrS-SpeMreB4. (**A**) Two-dimensionally averaged image of PrS-SpeMreB1 sheet at the condition of Fig. 1C from 881 particles. The subunit repeat is estimated to be 5.0 ± 0.2 nm. (**B–E**) Negative staining EM images of (**B**) 10 μM and (**C–D**) 30 μM PrS-SpeMreB1 WT and (**E**) 5 μM SpeMreB4 WT in (**C** and **E**) the standard buffer

and (**B** and **D**) that with the increasing KCl concentration of 500 mM in the (**B** and **E**) presence or (**C**–**D**) absence of 2 mM MgATP.

Fig. S4. Sedimentation assays of PrS-SpeMreB1 variants. For gel images, protein size standards were visualized in Lane M with the molecular masses beside each band. The band positions of prey and bait in gel images of co-sedimentation assays are indicated by P and B, respectively. (**A**) Co-sedimentation assays of 3 μM SpeMreB5 WT and 1 μM to 5 μM PrS in the presence or absence of 2 mM ATP. (**B**) Co-sedimentation assays of 3 μM SpeMreB5 WT and 0 μM to 10 μM PSMB1v WT (top), PrS-SpeMreB1 WT (middle), or PrS (bottom) in the presence of 2 mM ATP. Pellet amounts of SpeMreB5 were estimated and summarized in Fig. 3B. (**C**–**D**) Co-sedimentation assays of 3 μM SpeMreB5 WT and 0 μM to 6 μM PrS-SpeMreB1 WT (top), PSMB1v WT (second top), PrS-SpeMreB1 E275R (second bottom), or PrS (bottom) in the presence of 2 mM (**C**) ADP or (**D**) AMPPNP. Pellet amounts of SpeMreB5 were estimated and summarized in Fig. 3B. (**E**) Liposome binding assays of 2 μM PrS-SpeMreB1 WT in the presence of 1 mM liposomes composed of 100% DOPG, DOPC, SM, or CL. Negatively charged lipids (DOPG and CL) are indicated by red characters. (**F**) The surface representation of the full-length SpeMreB1 monomer modeled by AlphaFold2 (1). The Coulombic electrostatic potential is indicated by the color gradient from blue (+10 kcal/mol/e) to red (−10 kcal/mol/e) via white (0 kcal/mol/e). Subdomains IA, IB, IIA, and IIB are indicated at the corresponding positions of the structure. The side view of the membrane binding region on most MreBs (2,3) is shown on the right beside the front view on the left.

Fig. S5. Behavior of syn3B cells expressing an PrS-SpeMreB1. (**A**) Time-lapse phase-contrast microscopy images of a representative syn3B cell taken every 0.3 s. Shown is the cell co-expressing PrS-SpeMreB1 WT and SpeMreB5 WT. Time zero indicates the start of image acquisition. Scale bar: 5 μm. (**B**) Protein profiles of the syn3B cells co-expressing PrS-SpeMreB1 WT and SpeMreB5 WT. The control syn3B cells carrying antibiotic selection marker (none), those expressing SpeMreB1 WT and SpeMreB5 WT (SpeMreB5-SpeMreB1), and purified PrS-SpeMreB1 (PrS-SpeMreB1 (purified)) were loaded onto the same gel as references. Protein size standards are shown in lane M with the molecular masses of each band on the left side. The bands of SpeMreB1 and SpeMreB5 are marked as 1 and 5, respectively. The band most likely to PrS-SpeMreB1 expressed in the syn3B cells is indicated by a yellow triangle.

Movie S1. Combined real-time movies (10 s) of three syn3B strains collected from phase contrast. The strain names are shown at the top left of each panel. The scale bar indicates the bottom of the right panel.

Movie S2. Real-time movie (10 s) of syn3B cells expressing PrS-SpeMreB1 WT and SpeMreB5 WT collected from phase contrast. The scale bar indicates the bottom of the right panel.

Supplementary data 1. Nucleotide sequence of multiple cloning sites in pCold-PrS. Sequences of PrS, 6xHis-tag, factor Xa cleavage site, NdeI site, and BamHI site are shown in gray, blue, brown, purple, and red, respectively. The 5’- and 3’-ends are respectively connected with 5’- and 3’-UTR of a cold shock gene, *cspA*.

ATGAATCATAAAGTGCATCATCATCATCATCACATGGCAAATATTACCGTTTTCTATAA
CGAAGACTTCCAGGGTAAGCAGGTCGATCTGCCGCCTGGCAACTATACCCGCGCCCA
GTTGGCGGCGCTGGGCATCGAGAATAATACCATCAGCTCGGTGAAGGTGCCGCCTGG

CGTGAAGGCTATCCTGTACCAGAACGATGGTTTCGCCGGCGACCAGATCGAAGTGGT
GGCCAATGCCGAGGAGTTGGGCCCGCTGAATAATAACGTCTCCAGCATCCGCGTCAT
CTCCGTGCCCGTGCAGCCGCGCATGGCAAATATTACCGTTTTCTATAACGAAGACTTC
CAGGGTAAGCAGGTCGATCTGCCGCCTGGCAACTATACCCGCGCCCAGTTGGCGGCG
CTGGGCATCGAGAATAATACCATCAGCTCGGTGAAGGTGCCGCCTGGCGTGAAGGCT
ATCCTCTACCAGAACGATGGTTTCGCCGGCGACCAGATCGAAGTGGTGGCCAATGCC
GAGGAGCTGGGTCCGCTGAATAATAACGTCTCCAGCATCCGCGTCATCTCCGTGCCG
GTGCAGCCGAGGGGTACC**ATTGAAGGCCGCCATATG**GTCGACCTCGAG**GGATCC**CGTG
GTGAAATC**CATCACCATCACCATCAC**TAATCTAGATAGGTAA

Supplementary data 2. Amino acid sequences and accession IDs of SMreB1 and SMreB4 used in expression experiments (Fig. S1B–C). Accession IDs are summarized for sequences deposited in GenBank. The amino acid sequences are summarized for the other sequences.

>SpeMreB1: WP_047791301.1
>SpeMreB4: WP_047792066.1
>SmerMreB1: WP_004028643.1
>SmerMreB4: WP_004028011.1
>SkuMreB4: WP_053390367.1
>SapiMreB4: WP_023790308.1
>SlitMreB1: WP_075058542.1
>AnCitMreB1/4
MALFNSKKPTFVSMDLGTANTLVYVSGSGIVYNEPSIVAYKIKENRIIAVGNEAYKMIGKGNKS
IRIVRPMVDGVITDIRATEAQLRYIFNKLRISKQLKNSIMLLACPSVITELEKNALKKIAMNLGAD
KVFVEEEVKMAALGGGVDIYKPAGNLVVDMGGGTTDIAVLASGDIVLSKSVKVAGNYLNDEI
QKFIRSQYGLEIGIKTAEQIKIEIGSLAKYPDERKMKVYGRDVVSGLPREIEVTPEEIREVLKVPVS
RIIDLTVQVLEETPPELAGDIFRNGITICGGGALIKGIDKYFEDTLQLPAKIGEQPLLAVINGTKKF
ESDIYDILRQEHMHTKELNY

>AnSMreB1/4
MAKKKSKKPTFVSMDLGTANTLVYISGQGIVYNEPSIVAYKIKENKIIAVGEEAYKMIGKGNKN
IRIVRPMVDGVITDIRATEAQLRYIFNKLRISKTLKNSIMLLACPSVITELEKNALKKIAMNLGAD
KVFVEEEVKMAALGGGVNIYAPTGNLVVDMGGGTTDIAVLASGDIVLSKSIKVAGNYLNDEIQ
KFIRSQYGLEIGIKTAEQIKINIGSLAKYPDERKMKVYGRDVVSGLPREIEITPEEIREVLKVPVSRI
IDLTVQVLEETPPELAGDIFRNGITICGGGALIKGIDKYFEDTLQLPTKIGEQPLLAVINGTKKFES
DIYDILKEEHNHTKELNY

References

1. Mirdita, M., Schütze, K., Moriwaki, Y., Heo, L., Ovchinnikov, S., and Steinegger, M. (2022) ColabFold: making protein folding accessible to all. *Nat Methods* **19**, 679-682
2. Pande, V., Mitra, N., Bagde, S. R., Srinivasan, R., and Gayathri, P. (2022) Filament organization of the bacterial actin MreB is dependent on the nucleotide state. *J Cell Biol* **221**, e202106092
3. Salje, J., van den Ent, F., de Boer, P., and Löwe, J. (2011) Direct membrane binding by bacterial actin MreB. *Mol Cell* **43**, 478-487

Fig S1

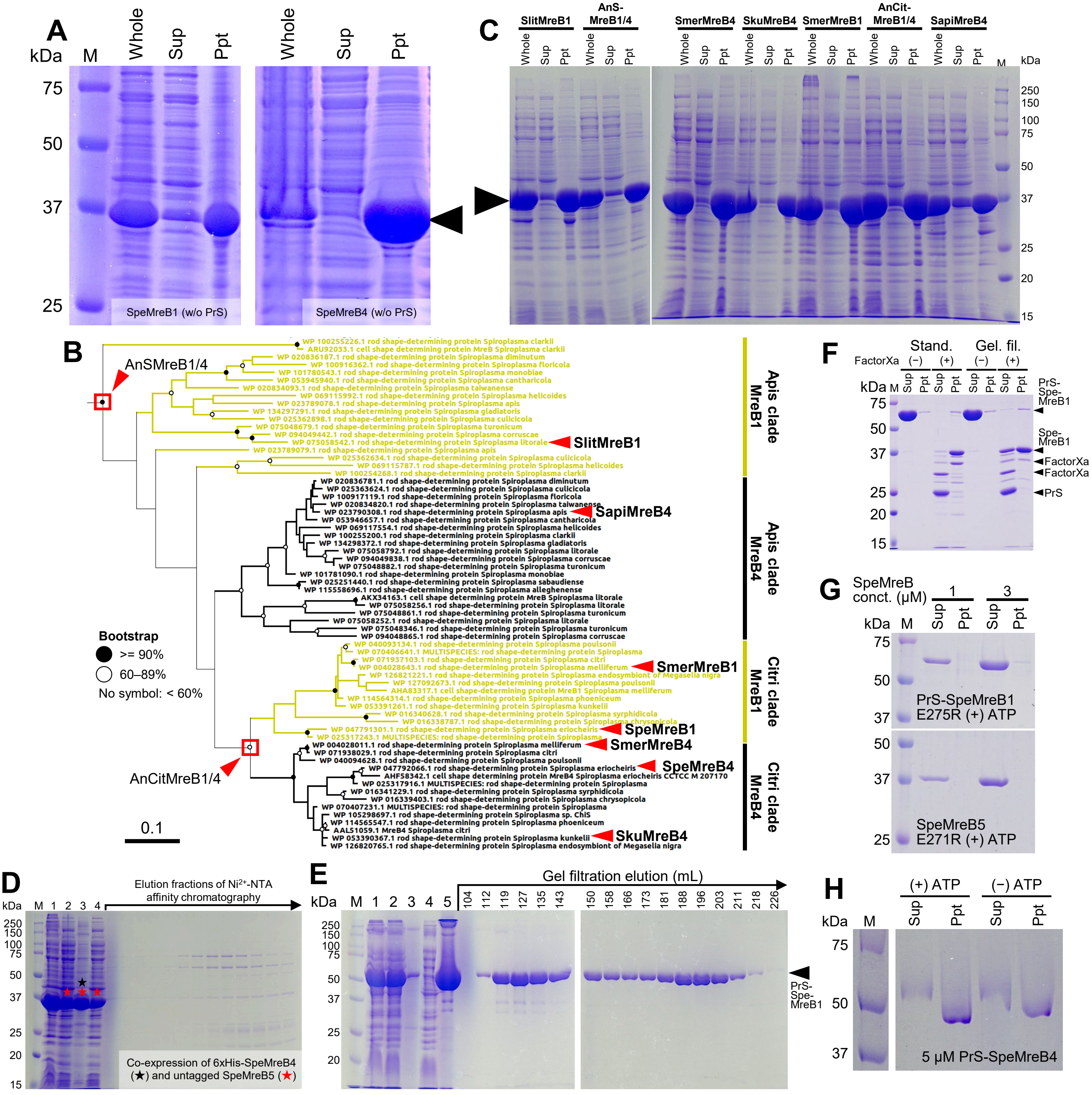

A
kDa
M
Whole
Sup
Ppt
75
50
37
25
SpeMreB1 (w/o PrS)
SpeMreB4 (w/o PrS)
C
SlitMreB1
AnS-MreB1/4
SmerMreB4
SkuMreB4
SmerMreB1
AnCit-MreB1/4
SapiMreB4
kDa
250
150
100
75
50
37
25
20
15
B
AnSMreB1/4
SlitMreB1
SapiMreB4
SmerMreB1
SpeMreB1
SmerMreB4
SpeMreB4
SkuMreB4
AnCitMreB1/4
Apis clade MreB1
Apis clade MreB4
Citri clade MreB1
Citri clade MreB4
Bootstrap
>= 90%
60–89%
No symbol: < 60%
0.1
F
FactorXa
Stand.
Gel. fil.
(−)
(+)
PrS-Spe-MreB1
Spe-MreB1
FactorXa
PrS
G
SpeMreB conct. (μM)
1
3
PrS-SpeMreB1 E275R (+) ATP
SpeMreB5 E271R (+) ATP
D
Elution fractions of Ni²⁺-NTA affinity chromatography
Co-expression of 6xHis-SpeMreB4 (★) and untagged SpeMreB5 (★)
E
Gel filtration elution (mL)
104
112
119
127
135
143
150
158
166
173
181
188
196
203
211
218
226
PrS-Spe-MreB1
H
(+) ATP
(−) ATP
5 μM PrS-SpeMreB4

Fig S2

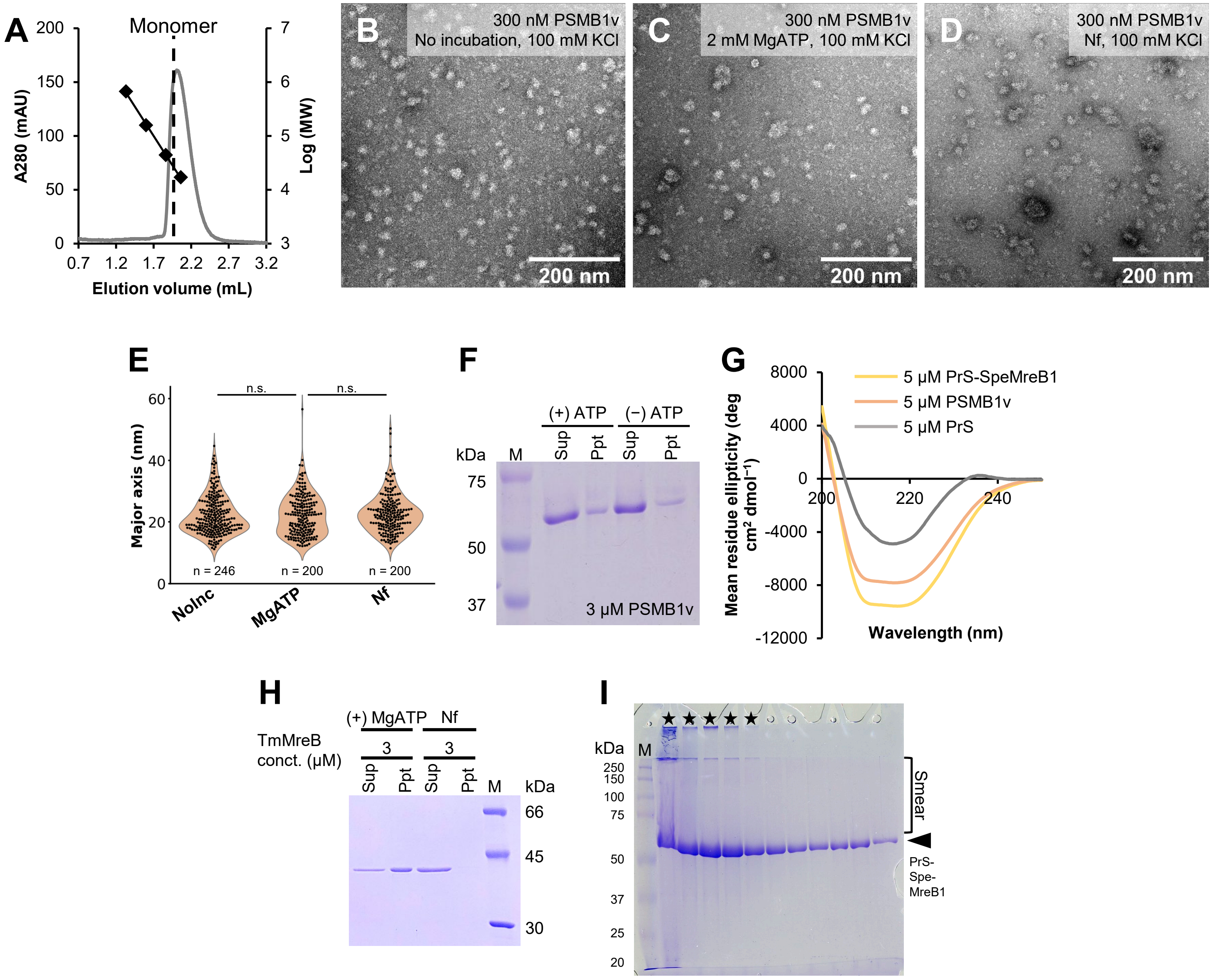
A
Monomer
A280 (mAU)
Log (MW)
Elution volume (mL)
B
300 nM PSMB1v
No incubation, 100 mM KCl
200 nm
C
300 nM PSMB1v
2 mM MgATP, 100 mM KCl
200 nm
D
300 nM PSMB1v
Nf, 100 mM KCl
200 nm
E
Major axis (nm)
n.s.
n.s.
n = 246
n = 200
n = 200
NoInc
MgATP
Nf
F
(+) ATP
(−) ATP
kDa
M
Sup
Ppt
Sup
Ppt
75
50
37
3 µM PSMB1v
G
5 µM PrS-SpeMreB1
5 µM PSMB1v
5 µM PrS
Mean residue ellipticity (deg cm² dmol⁻¹)
Wavelength (nm)
H
(+) MgATP
Nf
TmMreB
conct. (µM)
3
3
Sup
Ppt
Sup
Ppt
M
kDa
66
45
30
I
kDa
M
250
150
100
75
50
37
25
20
Smear
PrS-
Spe-
MreB1

Fig S3

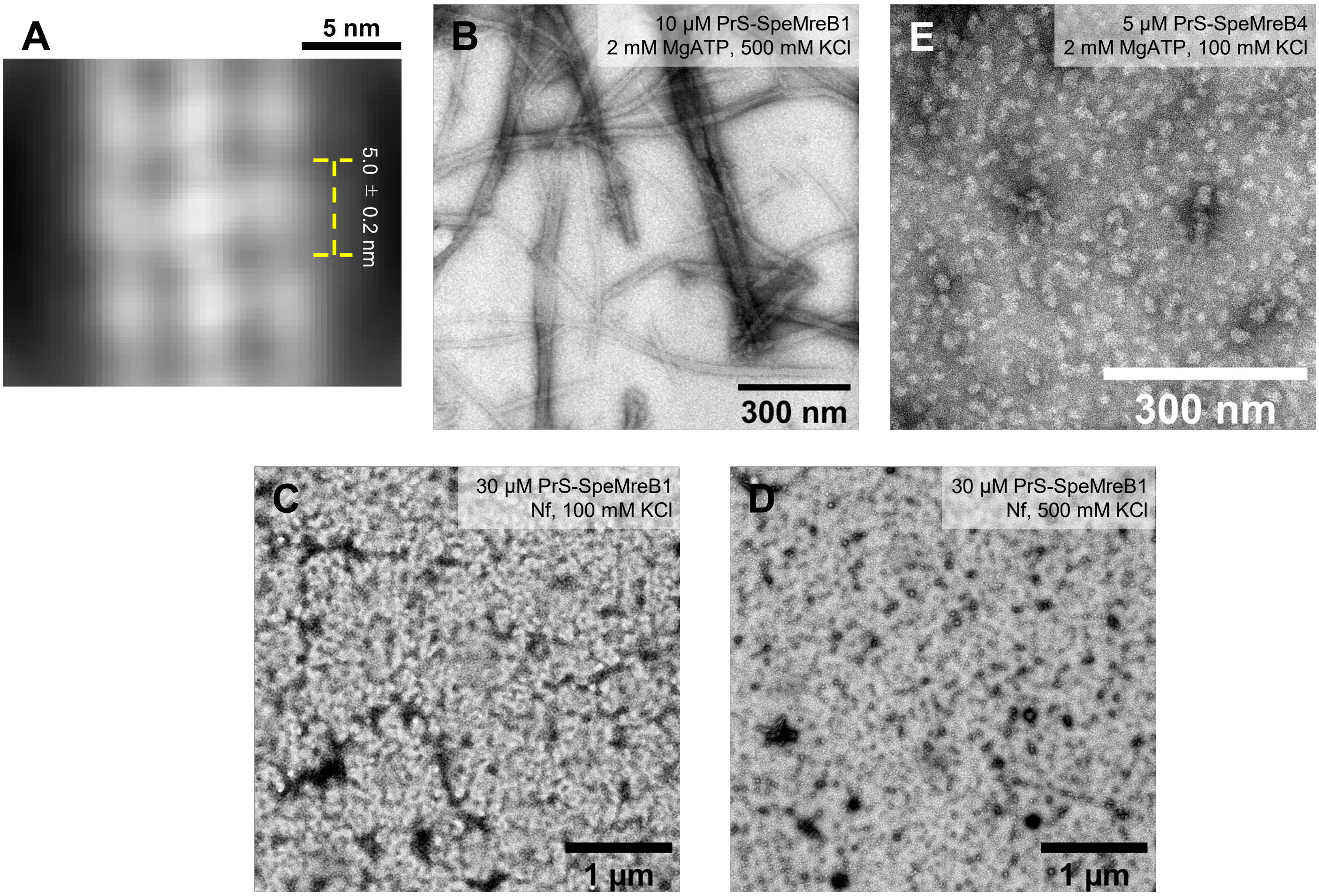
A
5 nm
5.0 ± 0.2 nm
B
10 µM PrS-SpeMreB1
2 mM MgATP, 500 mM KCl
300 nm
E
5 µM PrS-SpeMreB4
2 mM MgATP, 100 mM KCl
300 nm
C
30 µM PrS-SpeMreB1
Nf, 100 mM KCl
1 µm
D
30 µM PrS-SpeMreB1
Nf, 500 mM KCl
1 µm

Fig S4

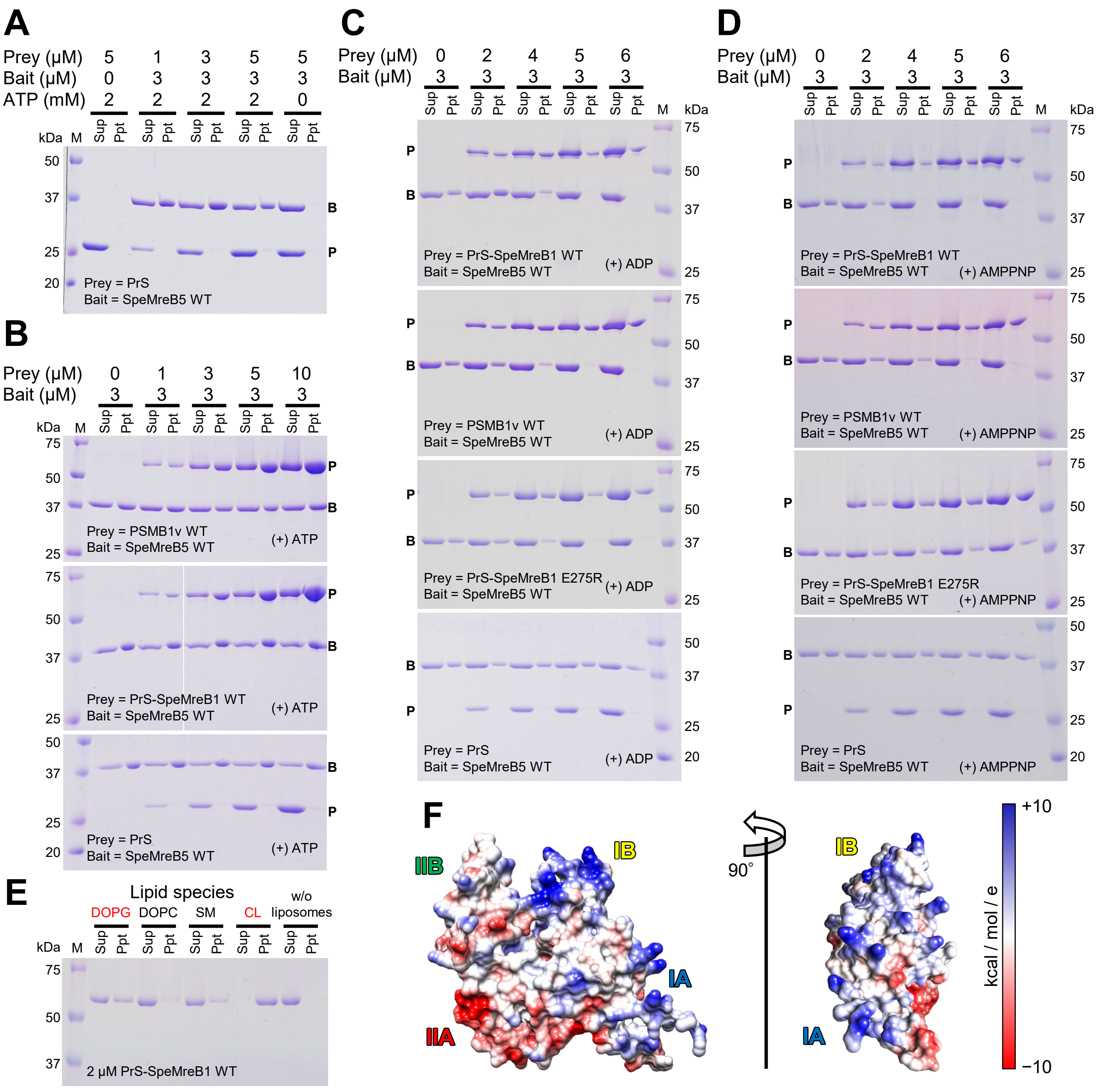
A
Prey (μM) 5 1 3 5 5
Bait (μM) 0 3 3 3 3
ATP (mM) 2 2 2 2 0
Prey = PrS
Bait = SpeMreB5 WT
B
Prey (μM) 0 1 3 5 10
Bait (μM) 3 3 3 3 3
Prey = PSMB1v WT
Bait = SpeMreB5 WT
(+) ATP
Prey = PrS-SpeMreB1 WT
Bait = SpeMreB5 WT
(+) ATP
Prey = PrS
Bait = SpeMreB5 WT
(+) ATP
C
Prey (μM) 0 2 4 5 6
Bait (μM) 3 3 3 3 3
Prey = PrS-SpeMreB1 WT
Bait = SpeMreB5 WT
(+) ADP
Prey = PSMB1v WT
Bait = SpeMreB5 WT
(+) ADP
Prey = PrS-SpeMreB1 E275R
Bait = SpeMreB5 WT
(+) ADP
Prey = PrS
Bait = SpeMreB5 WT
(+) ADP
D
Prey (μM) 0 2 4 5 6
Bait (μM) 3 3 3 3 3
Prey = PrS-SpeMreB1 WT
Bait = SpeMreB5 WT
(+) AMPPNP
Prey = PSMB1v WT
Bait = SpeMreB5 WT
(+) AMPPNP
Prey = PrS-SpeMreB1 E275R
Bait = SpeMreB5 WT
(+) AMPPNP
Prey = PrS
Bait = SpeMreB5 WT
(+) AMPPNP
E
Lipid species
DOPG DOPC SM CL w/o liposomes
2 μM PrS-SpeMreB1 WT
F
IIB IB IA IIA
90°
+10
kcal / mol / e
−10

Fig S5

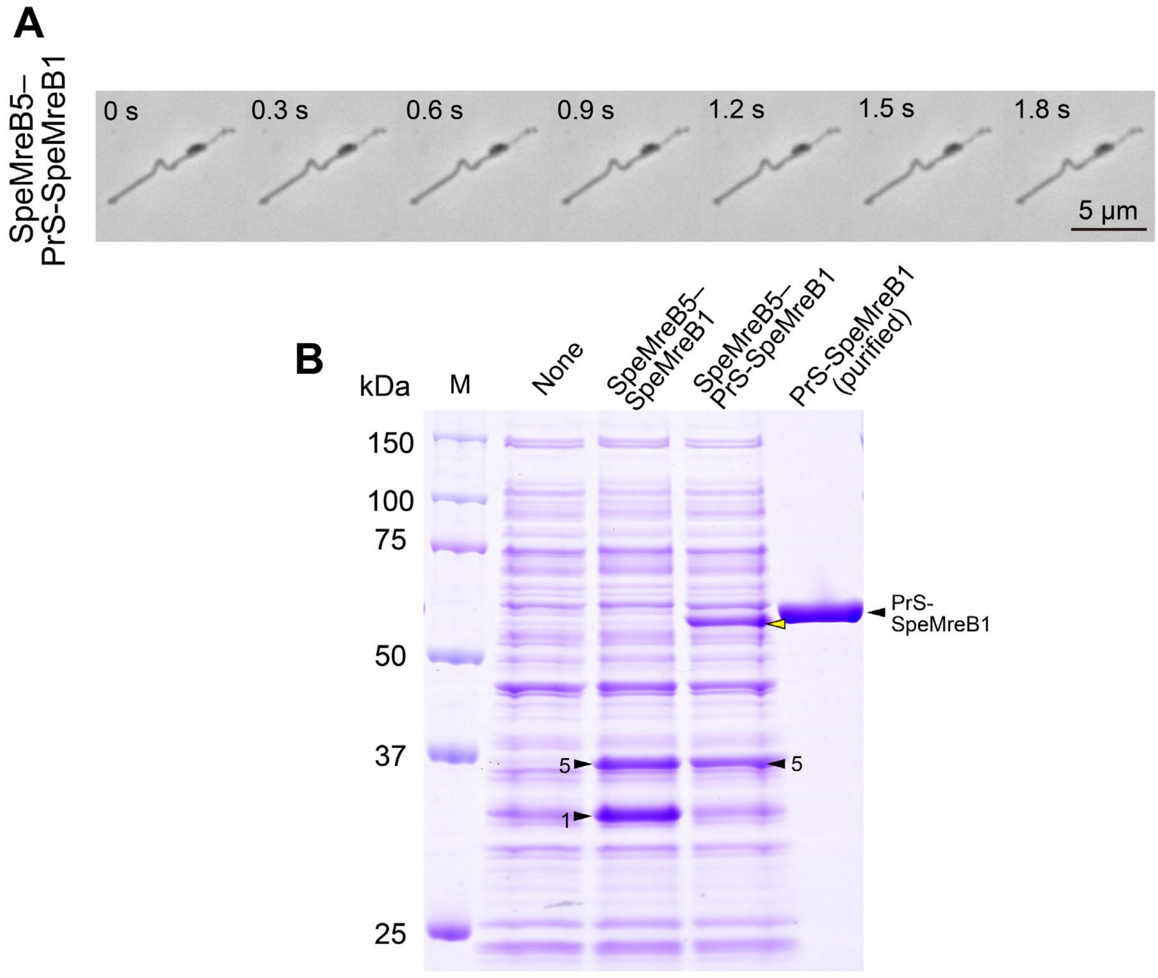
A
SpeMreB5–
PrS-SpeMreB1
0 s
0.3 s
0.6 s
0.9 s
1.2 s
1.5 s
1.8 s
5 µm
B
kDa
M
None
SpeMreB5–
SpeMreB1
SpeMreB5–
PrS-SpeMreB1
PrS-SpeMreB1
(purified)
150
100
75
50
37
25
PrS-
SpeMreB1
5
1
5
